# Electrochemistry and Stability of 1,1′-Ferrocene-Bisphosphonates

**DOI:** 10.1021/acsomega.2c07234

**Published:** 2023-03-09

**Authors:** Melissa Egger, Ingo Koehne, Dominik Wickenhauser, Werner Schlemmer, Stefan Spirk, Rudolf Pietschnig

**Affiliations:** † Institute of Bioproducts and Paper Technology, Graz University of Technology, Inffeldgasse 23, 8010 Graz, Austria; ‡ Institute of Chemistry and Center for Interdisciplinary Nanostructure Science and Technology (CINSaT), University of Kassel, Heinrich-Plett-Str. 40, 34132 Kassel, Germany

## Abstract

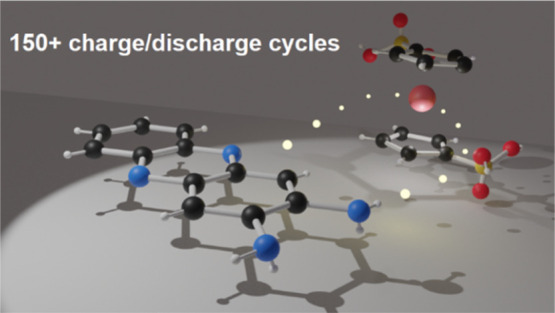

Here, we investigate the electrochemical properties and stability of 1,1′-ferrocene-bisphosphonates in aqueous solutions. ^31^P NMR spectroscopy enables to track decomposition at extreme pH conditions revealing partial disintegration of the ferrocene core in air and under an argon atmosphere. ESI-MS indicates the decomposition pathways to be different in aqueous H_3_PO_4_, phosphate buffer, or NaOH solutions. Cyclovoltammetry exhibits completely reversible redox chemistry of the evaluated bisphosphonates, sodium 1,1′-ferrocene-bis(phosphonate) (**3**) and sodium 1,1′-ferrocene-bis(methylphosphonate) (**8**), from pH 1.2 to pH 13. Both the compounds feature freely diffusing species as determined using the Randles-Sevcik analysis. The activation barriers determined by rotating disk electrode measurements revealed asymmetry for oxidation and reduction. The compounds are tested in a hybrid flow battery using anthraquinone-2-sulfonate as the counterside, yielding only moderate performance.

## Introduction

**Scheme 1:**
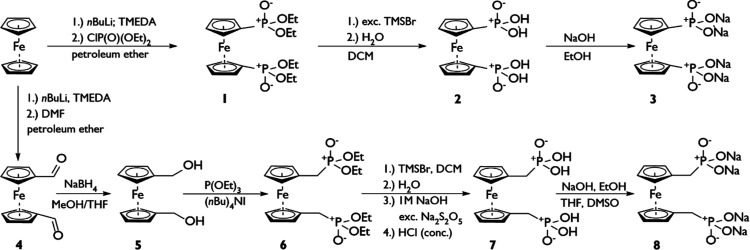
Synthesis of Catholytes **3** and **8**

Policymakers across the world have recognized the challenges associated with climate change, requiring a dramatic reduction of CO_2_ emissions and dependency on fossil resources.^1,2^ To address these problems, green and renewable energy conversion in wind and solar parks has been a major focus in the recent past. The increasing share of renewable energy in electricity supply still requires back-up systems to compensate for fluctuations in green energy supply due to weather conditions and season.^3^ The availability of large-scale energy-storage systems with low carbon footprint and high efficiency is, however, limited. Pumped hydropower and compressed air storage can provide sustainable energy in the GW range with high efficiency.^4^ However, there is lack of suitable sites for these technologies to significantly affect the challenges associated with green energy supply. In addition, most of commercial battery technologies rely on depletable sources, with lithium-ion batteries (LIBs) being the most prominent example. Although cost competitive in mobility and consumer electronics applications, LIBs still feature several disadvantages for stationary storage applications, such as self-discharge, limited cycle life, flammability, and availability of critical raw materials. Redox-flow batteries (RFBs) are different from LIBs as they store energy in the form of dissolved redox-active species, allowing for an independent design of power and storage capacity.^5^ Similar to LIBs, most commercial RFBs rely on metals such as vanadium and zinc that act together with bromine as redox-active species causing significant environmental impact.^6−9^ Alternative redox-active species comprise organic molecules from renewable sources or iron compounds [e.g., K_4_FeCN_6_, ferrocene (Fc)] which have some drawbacks concerning stability of their redox states.^10−12^ Moreover, neat Fc exhibits poor solubility in aqueous media over the whole pH range, limiting its application in flow batteries. Therefore, introduction of hydrophilic, charged groups (e.g., SO_3_^–^) with or without alkyl spacers have been proposed to mitigate the solubility challenges while increasing the volumetric energy density.^8^ However, one of the main problems to design improved Fc-based flow battery electrolytes stems from the lack of information regarding potential decomposition pathways.^7,13^ A synthetic strategy to monitor changes is to attach phosphonate groups close to the metallocene. These groups enhance the solubility of ferrocene compounds over a wide pH range and, more importantly, they enable the analysis of the electrolytes using ^31^P NMR spectroscopy. To get more insights into the stability of Fc-based electrolytes, we have adapted and improved the synthesis of two known Fc-bisphosphonates: sodium 1,1′-ferrocene-bis(phosphonate) (**3**) and sodium 1,1′-ferrocene-bis(methylphosphonate) (**8**) (Scheme 1).^14,15^ The stability of these compounds at different conditions (pH values, air vs argon) is followed ex situ as well as in sandwich cells and a full flow battery cell using two different anolytes [2,3-diaminophenazine (DAP) and anthraquinone-2-sulfonate (AQS)].

## Results and Discussion

The preparation of the title compounds starts from Fc proceeding via a three- or five-step synthesis (Scheme 1). Compound **3** is prepared starting with lithiation and subsequent salt-metathesis with diethyl chlorophosphate to yield 1,1′-ferrocene-bis(diethylphosphonate) (**1**). Subsequent ester hydrolysis using McKenna conditions^16^ yields 1,1′-ferrocene-bis(phosphonic acid) (**2**).^14^ In the last step, compound **2** is deprotonated via 4 equiv of NaOH in EtOH resulting in pure **3** to precipitate as an orange-yellowish solid. To introduce methylene spacers at the Fc unit, 1,1′-ferrocenedicarboxaldehyde (**4**) is synthesized via lithiation of Fc and reaction with dry DMF. A subsequent reduction with NaBH_4_ yields 1,1′-ferrocenedimethanol (**5**) that is subjected to an alcohol-based Michaelis–Arbuzov reaction^17^ giving bisphosphonate ester 1,1′-ferrocene-bis-(diethyl-methylphosphonate) (**6**). From this precursor, 1,1′-ferrocene-bis(methylphosphonic acid) (**7**) again is accessed via McKenna reaction.^14,16^ Compound **7** shows a poorer solubility in EtOH than its congener **2**, so that a NaOH-mediated deprotonation had to be carried out in a mixture of EtOH, THF, and DMSO to access the methylene-bridged sodium 1,1′-ferrocene-bis(methylphosphonate) (**8**). The solution ^31^P NMR spectra show one signal for each compound (**1**, **2**, **6**, **7**) with characteristic chemical shifts ranging from 20.4 to 25.0 ppm, while compounds **3** and **8** resonate at 15.3 and 18.8 ppm in D_2_O at pH 7. The solubilities in demineralized water at room temperature are determined to be 0.84 mol/·L^−1^ (**3**) and 0.41 mol/·L^−1^ (**8**) which correspond to theoretical RFB capacities^8^ of 22.5 Ah/·L^−1^ (**3**) and 11.0 Ah/·L^−1^ (**8**), respectively (for details, see the Supporting Information).

**Figure 1:**
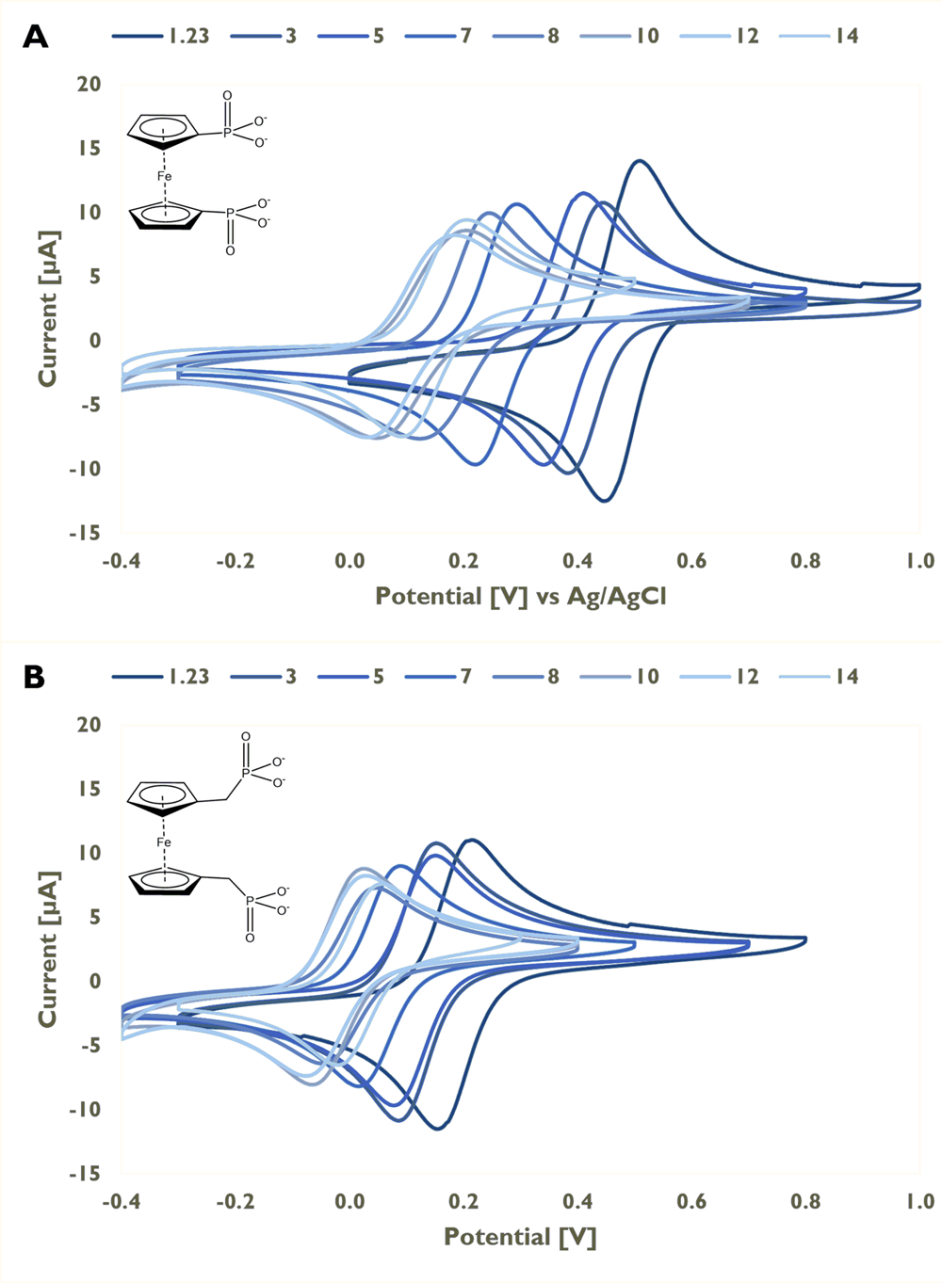
Evolution of the cyclic voltammograms as a function of pH value. (A) Sodium 1,1′-ferrocene-bis(phosphonate) (**3**); (B) sodium 1,1′-ferrocene-bis(methylphosphonate) (**8**). Scan rate: 100 mV·s^−1^; concentration: 0.5 mg·mL^−1^.

Both title compounds feature a fully reversible redox chemistry over the whole investigated pH range (1.23–14, Figure 1). In general, deprotonation of the phosphonic acid entails a cathodic shift of the oxidation potential of the adjacent ferrocene unit consistent with the results obtained for monosubstituted ferrocenylphosphonic acid and its sodium salts.^15^ The methylene spacer groups in **8** cause a significant potential shift over the whole pH range compared to compound **3** (e.g., at pH 1.23: **3**: 460 mV; **8**: 190 mV). The resulting Pourbaix plots (Supporting Information, Figures S33 and S34) show slight deviation from linearity over a pH range from 1.2 to 10; for higher pH values, the formation of a plateau is observed as reported for other Fc/Fc^+^ redox couples.^18^ For both compounds, there are deviations from linearity at pH 7 (and to some minor extent at pH 5), which is close to the p*K*_a2_ of the title compounds. The acidity of the phosphonate protons (particularly of compound **3**) is influenced by the Fc oxidation state. This can lead to a distortion of the ideally linear correlation between the E^0^ and pH values in pH ranges apart from the p*K*_a_ values. A similar behavior has been described for carboxylated Fc compounds in H_2_O/MeCN mixtures.^18^ The plot of the peak currents vs the square root of the scan rate (Supporting Information, Figures S35 and S36) shows a linear behavior over the investigated range (5–1000 mV·s^−1^) at constant pH. The observed peak currents decrease with increasing pH value at each scan rate, which could be caused by a change in viscosity at higher pH values.

**Figure 2:**
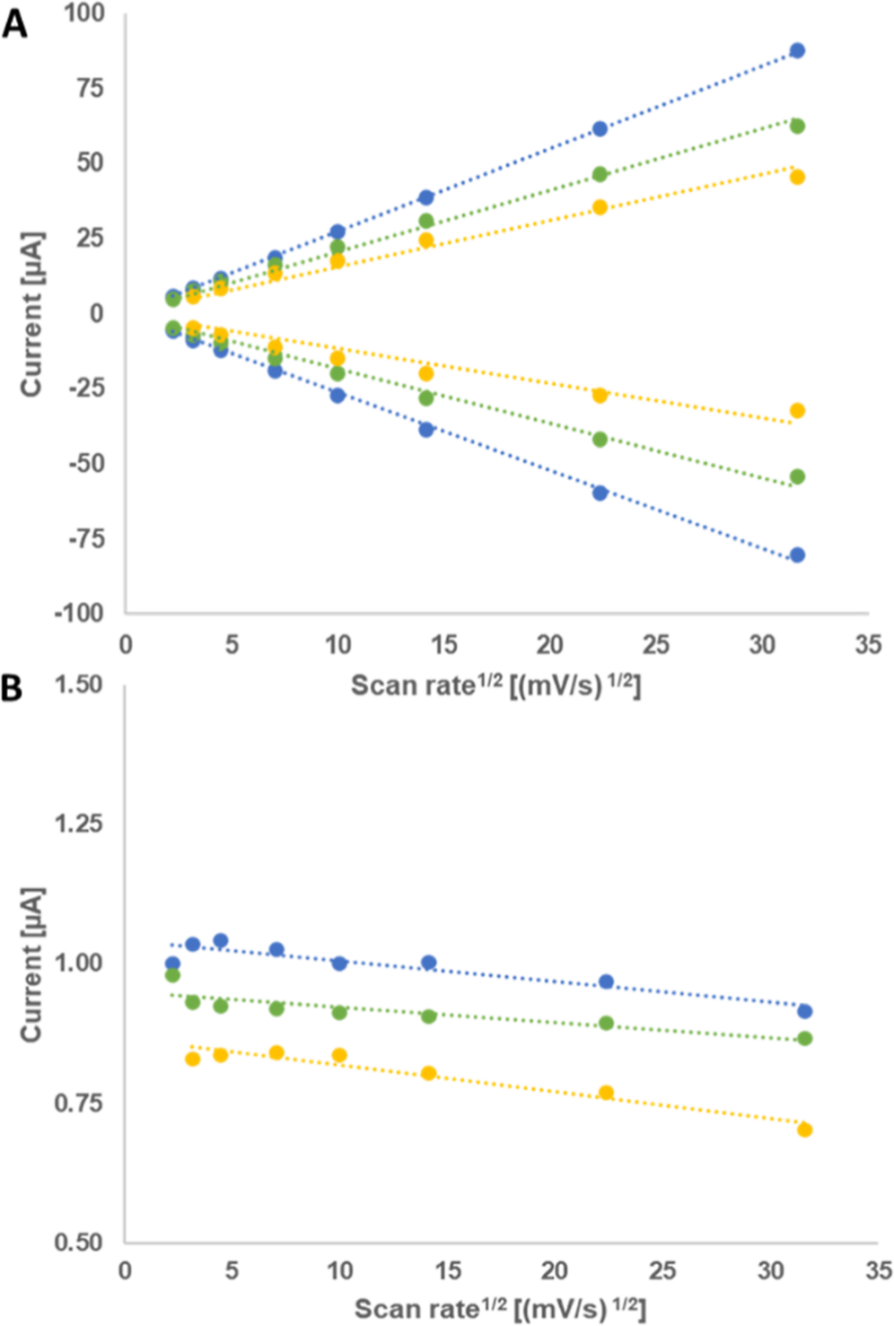
Randles–Sevcik plot for **8** at three different pH values with *c* ≈ 1.6 mg·mL^–1^. (A) Peak currents vs square root of the scan rate; (B) ratio of *I*_red_/*I*_ox_. Eight scan rates between 1000 and 5 mV s^–1^ were used. Blue: pH 1.23 (0.5 M H_3_PO_4_), green: pH 7 (0.5 M phosphate buffer solution), and yellow: pH 14 (1 M NaOH). pH 1.23: *I*_ox_ = 2.79 v^1/2^, *R*_2_ = 0.999. *I*_red_ = −2.65 v^1/2^, *R*_2_ = 0.999. pH 7: *I*_ox_ = 2.05 v^1/2^, *R*_2_ = 0.998. *I*_red_ = −1.81 v^1/2^, *R*_2_ = 0.995. pH 14: *I*_ox_ = 1.55 v^1/2^, *R*_2_ = 0.993. *I*_red_ = −1.16 v^1/2^, *R*_2_ = 0.977. (B) *I*_red_/*I*_ox_ vs square root of the scan rate. pH 1.23: 1.00 ± 0.04, pH 7: 0.92 ± 0.03, and pH 14: 0.80 ± 0.05.

For a better understanding of the processes occurring at the electrode interfaces, the diffusion kinetics and activation energies have been elucidated from specialized plots. Linear correlation between the peak current and the square root of the scan rate indicates that the electron transfer occurs in a freely diffusing species for all three solvent mixtures (Figure 2A). In phosphoric acid, the correlation between the peak current and the square root of the scan rate is highly linear but changes to a slightly curved shape for neutral and alkaline solutions, indicating a less reversible electron transfer.^19^ The slope also decreases with increasing pH value. The ratio of reductive and oxidative peak currents (Figure 2B) is close to 1 for pH 1.23 and 7, indicating a high reversibility, with a slight deviation observable at higher scan rates. At pH 14, the oxidative current is almost 20% higher than the reductive one even at low scan rates, suggesting a divergence from reversible behavior at this pH value.

**Figure 3:**
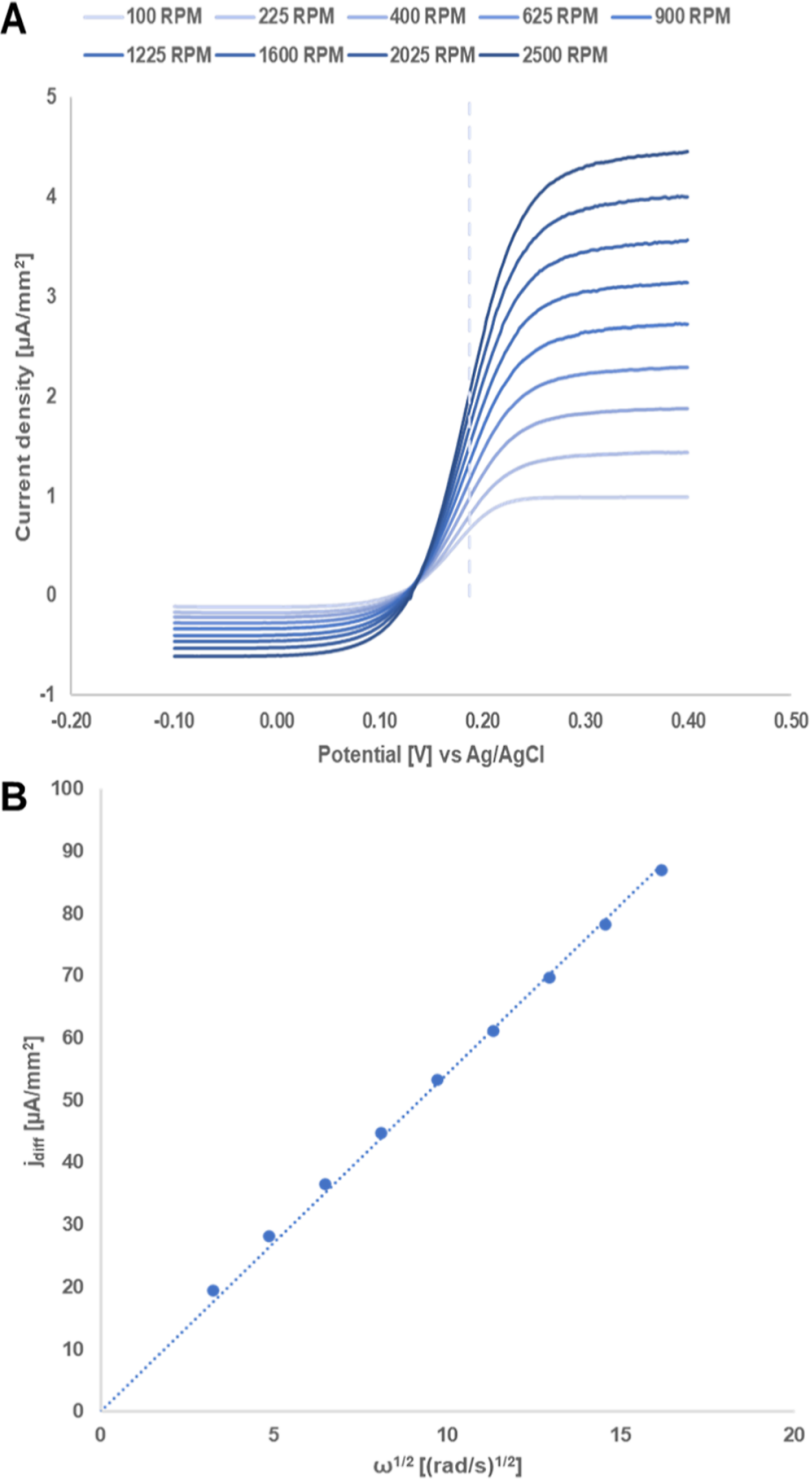
Levich experiment of **8** (0.76 mg·mL^–1^, 1.6 mM) in 0.5 M H_3_PO_4_ (pH 1.23). Cyclic voltammograms were recorded at 20 mV s^–1^, background corrected. (A) RDE measurements at nine rotation rates between 100 and 2500 RPM, and E0‘ marked by a dashed line (0.19 V). (B) Koutecky–Levich plot of diffusion limiting current (averaged current between 0.35 and 0.40 V) vs square root of rotation rate; lin. equiv: 1.75 x, *R*^2^ = 0.999.

The results of the rotating disk electrode (RDE) experiment (A) for **8** and the Levich plot (B) created from the averaged current at the plateau of the oxidative scan (between 0.35 and 0.40 V) are depicted in Figure 3. The diffusion coefficient is calculated from the slope under the assumption of a one-electron reaction with a kinematic viscosity of 0.95 mm^2^ s^–1^ for 0.5 M H_3_PO_4_ at 25 °C^20^ yielding 1.47 × 10^–6^ cm^2^ s^–1^, fitting well to the result from the Randles–Sevcik equation.

**Figure 4:**
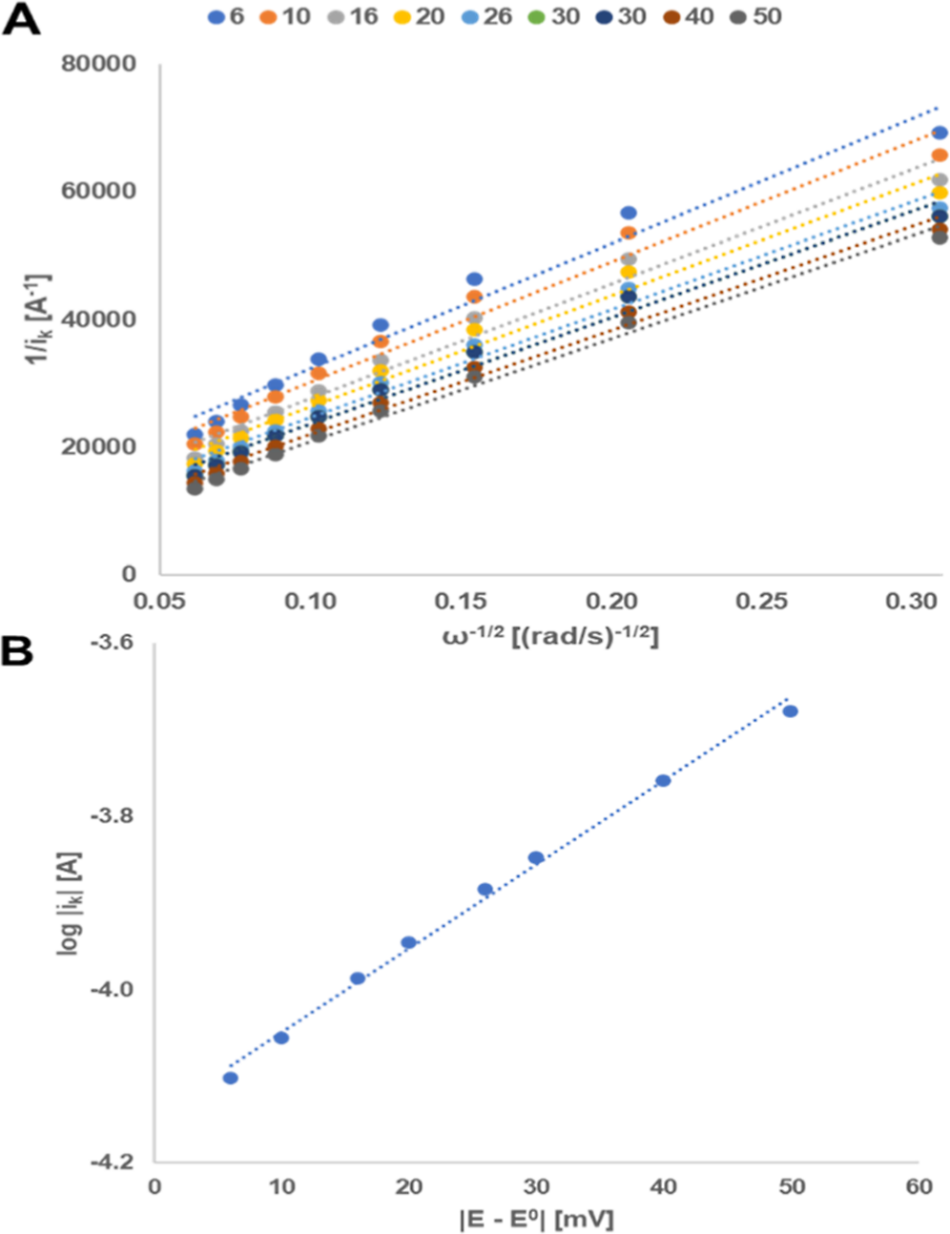
RDE experiment of **8** (0.76 mg·mL^–1^, 1.6 mM) in 0.5 M H_3_PO_4_ (pH 1.23). (A) Koutecky–Levich plot at overpotentials between 6 and 50 mV. (B) Tafel plot (logarithm of kinetically limited current vs overpotential), lin. equiv: 0.0097 *x* – 4.15, *R*^2^ = 0.995.

The Koutecky–Levich plot shows a series of parallel lines for each overpotential (Figure 4A), which allows us to plot a Tafel plot exhibiting good linearity (Figure 4B). The values for the kinetic rate constant and the symmetry factor are calculated from the Tafel plot, yielding *k*_0_ = 2.3 × 10^–3^ cm s^–1^ and α_ox_ = 0.57. The deviation of α from 0.5 again indicates that the activation barriers for reduction and oxidation are not fully symmetrical. A summary of kinetic data derived from Randles–Sevcik analysis and RDE is depicted in Table S5 (see the Supporting Information).

As we intend to test **3** and **8** as catholytes in RFBs, their stability must be thoroughly checked to ensure stable operation, either under ambient or inert conditions. The stability experiments comprised ^31^P NMR monitoring of **3** and **8** over a period of 3 weeks at three pH values under an air or argon atmosphere. For **3** under ambient conditions, we observe a downfield shift (21.2 ppm) in 0.5 M H_3_PO_4_ and a highfield shift (14.8 ppm) in 1 M NaOH compared to a solution in phosphate buffer (pH 7, 17.3 ppm). For compound **8**, downfield shifts to 21.0 ppm in 0.5 M H_3_PO_4_ and 20.2 ppm in phosphate buffer (pH 7) compared with a slight highfield shift to 18.2 ppm in 1 M NaOH are observed. The measurements do not reveal the formation of phosphorus-containing decomposition products for **3** or **8** in H_3_PO_4_ or phosphate buffer solutions. Instead, the observed line broadening in the case of H_3_PO_4_ solutions indicates the presence of paramagnetic Fe(II) high spin (h.s.) and/or Fe(III) low spin (l.s). species (Supporting Information, Figures S17, S18, S20, and S21). For **3** and **8** in NaOH solution, a new species with a ^31^P resonance of 5.2 ppm starts to emerge (most pronounced in the case of bisphosphonate **8**), while the signal intensities of **3** and **8** slowly start to decrease (Supporting Information, Figures S19 and S22). In the case of **8**, complete conversion of the starting material is observed after 3 weeks, while **3** is degraded only partially (ca. 20%). The chemical shift of 5.2 ppm of this decomposition product points at two candidates, namely, Cp-phosphonates or even Na_2_HPO_4_.^21^ As the chemical shift is identical for both compounds, it is likely that the dissolved Na_2_HPO_4_ is the main phosphorus-containing degradation product as also indicated by mass spectrometric analysis (Supporting Information, Figures S40–S42). To exclude the effects caused by oxidation or triplet oxygen, these experiments were repeated in an inert argon atmosphere. The measurements for **3** and **8** under argon again exhibit no formation of phosphorus-containing decomposition products in H_3_PO_4_ or phosphate buffer solutions (Supporting Information, Figures S23, S24, S26, and S27). For **3**, line broadening is even more excessive in argon than under ambient conditions, indicating a more effective formation of paramagnetic Fe(II) h.s. species under exclusion of oxygen. While decomposition of alkaline solutions in air yields a single side product detectable by ^31^P NMR spectroscopy, decomposition pathways in an argon atmosphere differ for **3** and **8**. Compound **8** does not show any signs of degradation in the ^31^P NMR spectra, featuring a single signal at 18.2 ppm which does not change over time (Supporting Information, Figure S28). In contrast, **3** exhibits a more complex decomposition behavior in the absence of air in NaOH solution. New signals are detected at 8.9 (day 2), 13.6 (day 4), and 7.9 ppm (day 7) (Supporting Information, Figure S25). The signal at 13.6 ppm might be attributed to a species like **3** but with a cleaved phosphonate side arm (also indicated by ESI-MS), while the other two signals most likely stem from iron-free Cp-phosphonates.

^1^H as well as ^13^C{^1^H} NMR investigations of the dried-out aging solutions of **3** and **8** from H_3_PO_4_, phosphate buffer or NaOH solutions show mostly, and as expected, only broad or no resonances due to formed paramagnetic side products (Supporting Information, Figures S44–S52). The observed signals can be assigned to the NMR solvent D_2_O or compounds **3** and **8**. Only in the case of the ^13^C{^1^H} spectra of **3** and **8** from the NaOH aging solutions, a single singlet resonance at 165.2 ppm of an unknown species can be observed. No hints of resonances around 6.50 ppm (^1^H) or 132.0 ppm (^13^C) that would indicate the presence of free cyclopentadiene are found.

With the exception of **8** in H_3_PO_4_, only minor amounts of precipitate formed over 3 weeks in the H_3_PO_4_ or buffer solutions in air (Supporting Information, Tables S1 and S2). In contrast, from solutions of **3** and **8** in NaOH, reddish-orange precipitates start to separate within 24 h that increase with advancing aging time. Just like for the H_3_PO_4_ and phosphate buffer solutions under ambient conditions, significant amounts of a precipitate only form for **8** in H_3_PO_4_ under argon within the considered period. In contrast, the NaOH solutions of **3** and **8** show a green-brownish to reddish-orange precipitate that again intensifies over time (Supporting Information, Tables S1 and S2). The formed precipitates imply the formation of iron hydroxide/oxides in all cases which was confirmed by IR spectroscopy.^22^ Absorption bands at 3400, 3300, and 1667 cm^–1^ can be assigned to different types of iron oxides [e.g., Fe_2_O_3_ or FeO(OH)]. Only in the case of the precipitate from **3** in NaOH, additional bands at 1235 cm^–1^ (P=O) and 1050 cm^–1^ (P–O) can be assigned to the presence of a phosphonate or phosphate species (Supporting Information, Figures S30–S32). In conclusion, catholytes **3** and **8** seem to be most stable in a phosphate buffer solution at pH = 7 showing no formation of phosphorus-containing decomposition products and the least precipitation or disturbance by paramagnetic species within the considered period.

ESI-MS investigations of the filtrates of the ^31^P NMR samples reveal two different decomposition pathways. As supported by aggregates with *m*/*z* = 404.95 and 293.12 (**3**) or *m*/*z* = 426.95 (**8**) (Supporting Information, Figures S40–S42), decomposition in H_3_PO_4_ or phosphate buffer solutions proceeds via extrusion of the iron ion from the Fc moiety under ligand exchange^23^ and formation of P–O–P-bridged bisphosphonate derivatives carrying cyclopentadiene residues. In contrast, a successive degradation is indicated for **3** and **8** in NaOH solutions (Supporting Information, Figure S43). For **3**, an aggregate with *m*/*z* = 288.99 can be assigned to a species with a cleaved phosphonate side arm that supports the assignment of the ^31^P NMR resonance at 13.6 ppm. A peak with *m*/*z* = 185.06 corresponds to an Fc radical cation and thus a species with two cleaved side arms. The C–P bond cleavages most likely proceed via hydroxodearylation as proposed for a bond cleavage in an isoelectronic C–Si moiety.^24^ In the last step, the iron ion again is torn out from the Fc unit under ligand exchange followed by Cp-anion oxidation and subsequent Cp-radical coupling. In contrast to a proposed formation of dicyclopentadiene by Yang^23^ but in accordance with the initial proposal by Kealy and Pauson,^25^ this results in the observation of the dominating aggregate with *m*/*z* = 128.99 which can be assigned to either a dihydro fulvalene radical cation or a [fulvalene + H^+^]^+^ aggregate.

**Figure 5:**
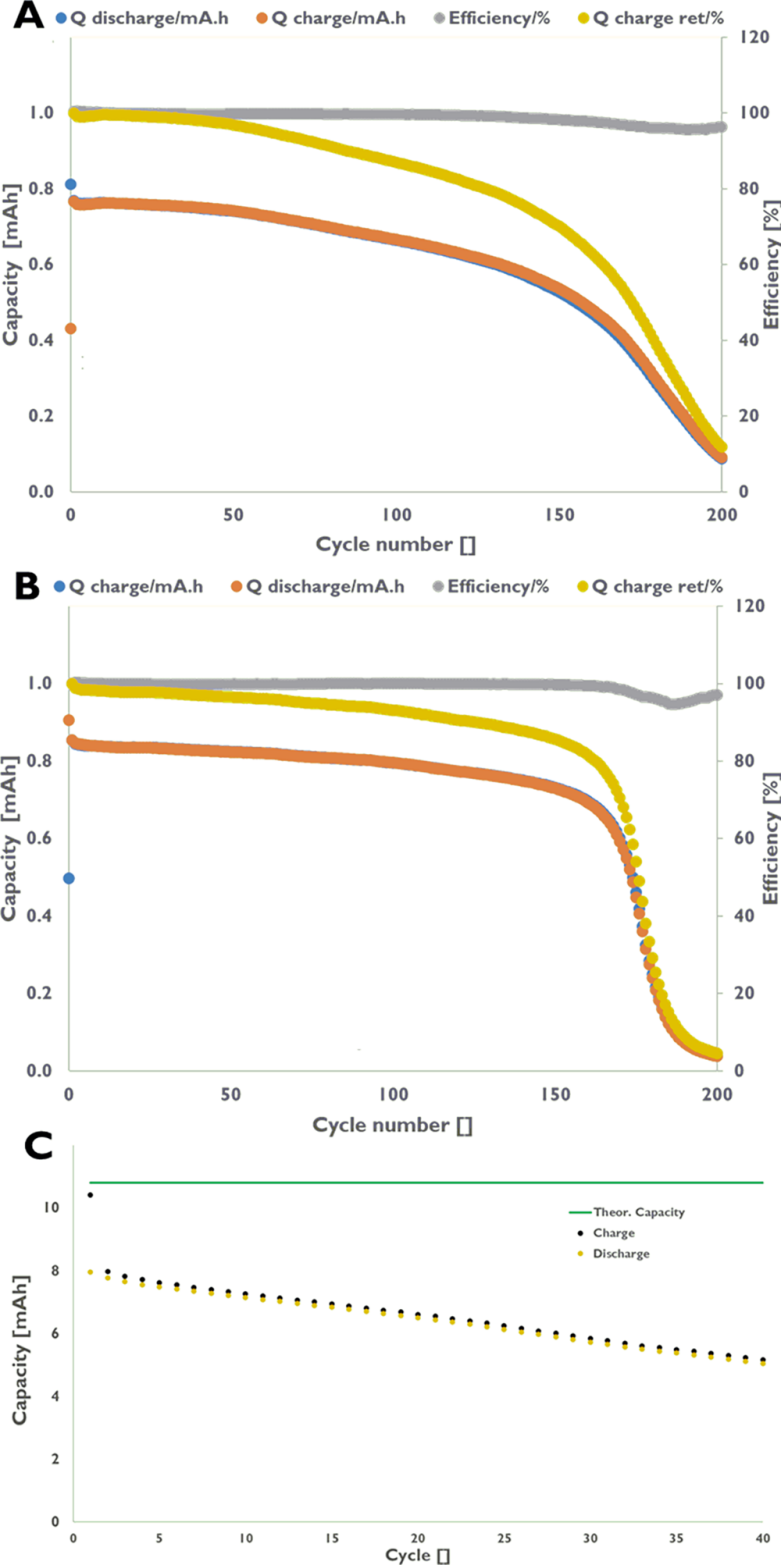
Potentiostatic cycling of the ferrocene derivates in a sandwich cell. (A) Charge–discharge curve for ferrocene-bis(phosphonate) (**3**) vs DAP. (B) Charge–discharge curve for ferrocene-bis(methylphosphonate) (**8**) vs DAP. Charge–discharge: 1.7 mg·mL^−1^ (3.9 mM for **3**, 3.7 mM for **8**) of the respective ferrocene derivate vs DAP in 0.5 M H_3_PO_4_ using a protonated Nafion 211 membrane. The cell was wrapped in a cling film during operation to avoid evaporation of the liquids. Potentiostatic charge/discharge was carried out at ±750 mV vs coulombic efficiency for 200 cycles. Charge capacity, discharge capacity, coulombic efficiency, and retained charge capacity (with respect to the second cycle) vs cycle number are shown. (C) Full RFB flow battery using **8** and AQS operated in 0.5 M H_3_PO_4_ using a Nafion 211 membrane under exclusion of oxygen at a current density of 12 mA·cm^−2^ at the solubility limit of **8** in 0.5 M H_3_PO_4_.

The chemical stability of **3** and **8** in phosphate buffer at pH 7 as well as in 0.5 M H_3_PO_4_ indicates potential use of these compounds in flow battery technology. To demonstrate their suitability, we assembled on the one hand sandwich cells (Supporting Information, Figures S38 and S39) using **3** and **8** as catholytes. The gravimetric capacity for compounds **3** and **8** in the tetra sodium form is 222.8 C·g^–1^/61.89 mA·h·g^–1^ and 208.9 C·g^–1^/58.02 mA·h·g^–1^, respectively. We cycled **3** and **8** against DAP and AQS, both commercially available compounds, which are well soluble in acidic media. At the chosen conditions, DAP and AQS are stable and do not decompose. DAP shows good performance in the sandwich cell for more than 50 cycles without any significant capacity loss during cycling. After 50 cycles, a slow decay is observed for the system running with **3**/DAP and yields ca. 70% capacity retention after 150 cycles (Figure 5A). The system operating with **8**/DAP is more stable (86% after 150 cycles) than **3**/DAP (Figure 5B). We anticipate that the loss of capacity after ca. 150 cycles can be attributed to imperfections in sealing the sandwich cells leading to dried-out electrodes. However, full flow battery systems could not be successfully tested with DAP as the anolyte as the capacity dropped to zero within a few cycles. With AQS as the anolyte, a flow battery could be run successfully but with moderate performance (Figure 5C). A large drop in capacity (20%) was observed after the first discharge cycle. Afterward, a slow decay was observed (0.7%/per cycle), leading to a capacity retention of ca. 48% after 40 cycles. We can exclude major cross diffusion as the cyclic voltammograms of the anolyte and catholyte solutions did not show any contaminations, while for DAP, and to a smaller extent also AQS, an enrichment of the species on and/or in the membrane was observed, potentially limiting proton transfer. In addition, the asymmetry of the activation in terms of oxidation and reduction is reflected on the cell level in both sandwich and, to a lesser extent on, flow battery cells and also contributes to lower charge-capacity retention for the chosen systems.

The results on flow battery and sandwich cells demonstrate that the decomposition pathways for electrochemically active molecules need to be thoroughly investigated to design novel energy-storage systems. This also involves considerations of interactions with the battery components and here, particularly, the membrane.

## Materials and Methods

### General Information

All manipulations involving air- and moisture-sensitive compounds were carried out under an argon atmosphere using Schlenk techniques or handled in an argon glovebox. Solvents were dried over Na or K metal or Na/K alloy and were used freshly distilled. Starting materials were purchased commercially and were used as received, unless stated otherwise. Compounds **1**, **2**, and **7** were prepared according to procedures by W. Henderson et al. that have been modified and improved.^14^ The reaction conditions for an alcohol-based Michaelis–Arbuzov reaction for compound **6** are obtained from Han et al.^17^ Filtering of moisture-sensitive compounds was carried out with self-made filter cannulas assembled from Whatman fiberglass filters (GF/B, 25 mm), which were applied with Teflon tape to Teflon cannulas. Flash chromatography was performed with an Interchim PuriFlash XS 520Plus device using PF-30SIHP-F0020 or -F0040 columns. CV = column volumes. For TLC, precoated Macherey-Nagel Alugram Xtra SIL G/UV_254_ plates were used. NMR experiments were performed with Varian 400 or 500 MHz spectrometers, and the spectra were processed with MestReNova (v11.0.4–18998, Mestrelab Research S.L.). ^1^H and ^13^C NMR spectra were referenced relative to TMS using the residual solvent signals as the secondary reference, and ^31^P NMR spectra were referenced relative to 85% H_3_PO_4_.^26^ IR spectra were recorded with a diamond or germanium probe ATR IR spectrometer by Bruker. Elemental analyses were performed using a HEKAtech Euro EA-CHNS elemental analyzer. For analyses, the samples were prepared in tin cups with V_2_O_5_ as an additive to ensure complete combustion. ESI mass spectra were recorded on a Finnigan LCQDeca (ThermoQuest) or a MicrOTOF (Bruker Daltonics) device. For aging experiments, three solutions of different pH [0.5 M H_3_PO_4_, 1 M NaOH, and a phosphate buffer solution (pH = 7) consisting of Na_2_HPO_4_ (31.4 mmol) and NaH_2_PO_4_ (18.5 mmol) in H_2_O (100 mL)] were prepared. 3 mL solutions at each pH value using compounds **3** and **8** with *c* = 1.7 mg·mL^−1^ were prepared for the experiments under ambient conditions. For measurements under an argon atmosphere, the pH solutions were degassed via three freeze-pump-thaw cycles prior to use, and 0.5 mL solutions (*c* = 10 mg·mL^−1^) at each pH value using compounds **3** and **8** were prepared in screw-capped NMR tubes in an argon-filled glovebox. For locking and shimming, a sealed capillary with C_6_D_6_ was added to all NMR samples. Coupled and decoupled ^31^P NMR spectra were measured with 128 scans. The solubility of **3** and **8** in demineralized water was determined by producing an oversaturated solution of each compound at room temperature. The suspensions were filtered, the solvent from each filtrate was removed, and the residues were thoroughly dried and weighed. Theoretical capacity = solubility (M) × 96,485 (C·mol^−1^)/3,600 (C·A^−1^·h^−1^).^8^

## Supporting Information Available

Experimental procedures and NMR spectra, ^31^P NMR investigations of aging solutions of **3** and **8**, ^31^P NMR and IR spectra, Pourbaix and Randles–Sevcik plots, Sandwich cell experiments, ESI-MS spectra, diffusion, and kinetic parameters for DAP and Fc derivatives (PDF)
