# Supplementary material for: Electrochemistry and Stability of 1,1′-Ferrocene-Bisphosphonates
Source: ACS Omega. 2023 Mar 9;8(12):10899–905. doi: 10.1021/acsomega.2c07234 (PMC10061590; doi:10.1021/acsomega.2c07234)
Supplement: Supplementary file 1 — ao2c07234_si_001.pdf [file ao2c07234_si_001.pdf]

Supporting Information

Electrochemistry and stability of 1,1’-ferrocene-bisphosphonates

Melissa Egger, [a] Ingo Koehne, [b] Dominik Wickenhauser, [a] Werner Schlemmer, [a] Stefan Spirk [a*] and Rudolf Pietschnig [b*]

[a]: Institute of Bioproducts and Paper Technology, Graz University of Technology, Inffeldgasse 23, 8010 Graz, Austria.

[b]: Institute of Chemistry and Center for Interdisciplinary Nanostructure Science and Technology (CINSaT), University of Kassel, Heinrich-Plett-Str. 40, 34132 Kassel, Germany.

*Email: stefan.spirk@tugraz.at
pietschnig@uni-kassel.de

Content

Content 1

1. Experimental Procedures 2

   Sodium 1,1'-ferrocene-bis(phosphonate) (**3**) 2

   Sodium 1,1'-ferrocene-bis(methylphosphonate) (**8**) 3

2. NMR Spectra 5

   1,1'-Ferrocene-bis(diethyl phosphonate) (**1**) 5

   1,1'-Ferrocene-bis(phosphonic acid) (**2**) 6

   Sodium 1,1'-ferrocene-bis(phosphonate) (**3**) 7

   1,1'-Ferrocenedicarboxaldehyde (**4**) 8

   1,1'-Ferrocenedimethanol (**5**) 9

   1,1'-Ferrocene-bis(diethylmethylphosphonate) (**6**) 10

   1,1'-Ferrocene-bis(methylphosphonic acid) (**7**) 11

   Sodium 1,1'-ferrocene-bis(methylphosphonate) (**8**) 12

3. ^{31}P NMR Investigations of Ageing Solutions of 3 & 8 at Different pH values under Air & Argon..... 13

4. Stacked $^{31}P\{^{1}H\}$ NMR Spectra of Ageing Solutions of 3 & 8 at Different pH Values 16

5. IR-Spectra of the Precipitates from Solutions of 3 and 8 at Different pH Values 22

6. Pourbaix Plots 24

7. Randles-Sevcik Plots 25

8. Sandwich and Full Flow Cell Experiments 26

9. ESI-MS Spectra of the Filtrates of the ^{31}P-NMR Samples of 3 and 8 27

10. Diffusion and Kinetic Parameters for the DAP and Fc Derivatives 29

11. ^{1}H & $^{13}C\{^{1}H\}$ NMR Spectra of 3 & 8 after Aging Experiments under Argon in 0.5M H_3PO_4, Phosphate Buffer or 1M NaOH 30

1. Experimental Procedures

Sodium 1,1'-ferrocene-bis(phosphonate) (3)

1,1'-ferrocene-bis(diethyl phosphonate) (1). Freshly sublimed ferrocene (5.58 g, 30.0 mmol) was dissolved in a mixture of dry hexanes/pentanes (3:2, 120 mL) with the help of a little heat. *n*BuLi (24.1 mL, 2.5M in hexanes, 60.3 mmol), and then TMEDA (4.52 mL, 30.0 mmol) were added via syringe and the mixture was heated under reflux for 3 h. The orange suspension was cooled to RT and stirred overnight (16 h). A 250 mL Schlenk flask was charged with a mixture of dry hexanes/pentanes (3:2, 60 mL) and diethyl chlorophosphate (8.84 mL, 60.9 mmol) was added via syringe. To this solution, the dilithoferrocene suspension was added potion wise via a Teflon cannula at RT under vigorous stirring. A brown tar/oil separates upon addition. After completed addition, the mixture was stirred at RT for additional 30 min. The solvent was decanted, and H_2O (120 mL) and DCM (120 mL) were added to the residue. The phases were separated, and the aqueous phase was extracted with DCM (2 x 100 mL). The combined organic phases were washed with H_2O (2 x 100 mL), dried over $MgSO_4$, filtered, and the solvent was evaporated. The brownish residue was suspended in EtOAc (15 mL), filtered, the solvent volume was reduced to a minimum, and subjected to flash column chromatography (Silica; gradient over 18 CV from neat EtOAc to neat EtOH). Compound **1** was obtained as a brown oil (5.77 g, 12.6 mmol, 42%). ^{1}H NMR (400 MHz, $CDCl_3$): δ = 4.63–4.54 (m, 8H, H_{Ar}), 4.10 (m, 8H, CH_2CH_3), 1.33 (t, $^{3}J_{HH}$ = 7.0 Hz, 12H, CH_2CH_3) ppm; $^{13}C\{^{1}H\}$ NMR (100 MHz, $CDCl_3$): δ = 74.0 (d, 4C, $^{3}J_{PC}$ = 13.7 Hz, C_{Ar}), 73.0 (d, 4C, $^{2}J_{PC}$ = 15.2 Hz, C_{Ar}), 68.6 (d, 2C, $^{1}J_{PC}$ = 214 Hz, C_{Ar}), 61.9 (d, 4C, $^{2}J_{PC}$ = 6.0 Hz, CH_2CH_3), 16.57 (d, 4C, $^{3}J_{PC}$ = 6.4 Hz, CH_2CH_3) ppm; $^{31}P\{^{1}H\}$ NMR (202 MHz, $CDCl_3$): δ = 24.4 (s) ppm; IR (ATR) $\tilde{\nu}$ = 1242 (P=O), 1093 (P–OEt) cm^{-1}; MS (ESI+) *m/z* (%): 459.16 (45) $[M + H^+]^+$, 481.15 (95) $[M + Na^+]^+$, 490.13 $[M + MeOH + H^+]^+$, 939.35 $[2M + Na^+]^+$, Anal. Calcd for $C_{18}H_{28}FeO_6P_2$: C, 47.18; H, 6.16. Found: C, 45.58; H, 6.42. Deviation due to a contamination with DCM.

1,1'-ferrocene-bis(phosphonic acid) (2). 1,1'-ferrocenylene-bis(diethyl phosphonate) (4.58 g, 10.0 mmol) was dissolved in dry DCM (100 mL) and Me_3SiBr (10.6 mL, 80.0 mmol) was added drop wise via syringe at RT. The mixture was stirred at RT overnight (16 h). H_2O (2.52 mL, 140 mmol) was added, and the mixture was vigorously stirred for 30 min. The black aqueous phase was separated and the solvent from the organic phase was removed. Then, H_2O (60 mL) and a mixture of EtOAc/THF (2:1, 75 mL) were added to the residue, and the phases were separated. The aqueous phase was extracted with a mixture of EtOAc/THF (2:1, 5 x 25 mL), the combined organic phases were dried over $MgSO_4$, filtered, and the solvent was removed. The obtained dark yellow residue was suspended in acetone (20 mL), filtered over a Büchner funnel with suction, the filter cake was washed several times with small amounts of acetone, and dried. Compound **2** was obtained as a dark yellow solid (2.70 g, 7.81 mmol, 78%). ^{1}H NMR (400 MHz, DMSO-d_6): δ = 4.53 (s_{br}, 4H, H_{Ar}), 4.48 (s_{br}, 4H, H_{Ar}), 4.31 (s_{vbr}, 4H, OH) ppm; $^{13}C\{^{1}H\}$ NMR (100 MHz, DMSO-d_6): δ = 72.9 (d, 2C, $^{1}J_{PC}$ = 200 Hz, C_{Ar}), 72.4 (d, 4C, $^{2}J_{PC}$ = 15.2 Hz, C_{Ar}), 71.7 (d, 4C, $^{3}J_{PC}$ = 12.7 Hz, C_{Ar}) ppm; $^{31}P\{^{1}H\}$ NMR (202 MHz, DMSO-d_6): δ = 20.4 (s) ppm; MS (ESI+) *m/z* (%): 347.02 (100) $[M + H^+]^+$. Anal. Calcd for $C_{10}H_{12}FeO_6P_2$: C, 34.71; H, 3.50. Found: C, 34.93; H, 3.59.

Sodium 1,1'-ferrocene-bis(phosphonate) (3). In a 250 mL round-bottom flask, 1,1'-ferrocenylene-bis(phosphonic acid) (1,73 g, 5.00 mmol) was dissolved in EtOH (100 mL) with the help of a little heating. NaOH beads (804 mg, 20.1 mmol) were added, and the mixture was vigorously stirred at RT for 45 min. The formed orange precipitate was recovered via filtration over a pleated filter, washed with EtOH (2 x 20 mL), and air-dried. Compound **3** was obtained as an orange-yellowish solid (2.11 g, 4.85 mmol, 97%). ^{1}H NMR (400 MHz, D_2O): δ = 4.40 (s_{br}, 4H, H_{Ar}), 4.38 (s_{br}, 4H, H_{Ar}) ppm; $^{13}C\{^{1}H\}$ NMR

(100 MHz, D_2O): δ = 79.0 (d, 2C, $^{1}J_{PC}$ = 189 Hz, C_{Ar}), 71.5 (d, 4C, $^{2}J_{PC}$ = 13.5 Hz, C_{Ar}), 70.6 (d, 4C, $^{3}J_{PC}$ = 11.5 Hz, C_{Ar}) ppm; $^{31}P\{^{1}H\}$ NMR (202 MHz, D_2O): δ = 15.3 (s) ppm; IR (ATR) $\tilde{\nu}$ = 1252 (P=O), 1090 (P–ONa) cm^{-1}; MS (ESI+) *m/z* (%): 434.89 (100) $[M + H^+]^+$, 456.87 (30) $[M + Na^+]^+$; Anal. Calcd for $C_{10}H_8FeNa_4O_6P_2$: C, 27.68; H, 1.86. Found: C, 22.74; H, 3.88. Deviation due to a contamination with EtOH.

Sodium 1,1'-ferrocene-bis(methylphosphonate) (**8**)

1,1'-ferrocenedicarboxaldehyde (4). Freshly sublimed ferrocene (8.00 g, 43.0 mmol) was dissolved in a mixture of dry hexanes/pentanes (3:1, 180 mL) with the help of a little heat. *n*BuLi (34.4 mL, 2.5M in hexanes, 86.0 mmol), and then TMEDA (7.70 mL, 86.0 mmol) were added via syringe and the mixture was heated under reflux for 3 h. The orange suspension was cooled to RT and stirred overnight (16 h). A 500 mL Schlenk flask was charged with dry pentanes (40 mL), and dry DMF (6.62 mL, 86.0 mmol) was added via syringe. To this solution, the dilithioferrocene suspension was added potion wise via Teflon cannula at RT under vigorous stirring. After completed addition, the mixture was stirred at RT for 4 h. Then, H_2O (100 mL) was added, the phases were separated, and the organic phase was extracted with additional H_2O (100 mL) and brine (100 mL). The combined aqueous phases were extracted with EtOAc (3 x 100mL). The combined organic phases were dried over $MgSO_4$, filtered, and the solvent was removed. The reddish residue was suspended in Et_2O, filtered, and the solvent was removed again. The product is already pure enough for further reactions at this point. For analysis, a flash chromatography (Silica; 2 CV pentanes/EtOAc (8:2); 1 CV to reach DCM/EtOAc (9:1); 10 CV DCM/EtOAc (9:1)) gave compound **4** as a reddish-orange solid (7.54 g, 31.2 mmol, 72%). ^{1}H NMR (400 MHz, $CDCl_3$): δ = 9.94 (s, 2H, CHO), 4.87 (s, 4H, C_{Ar}), 4.66 (s, 4H, C_{Ar}) ppm; $^{13}C\{^{1}H\}$ NMR (100 MHz, $CDCl_3$): δ = 192.9 (s, 2C, CHO), 80.4 (s, 2C, C_{Ar}), 74.3 (s, 4C, C_{Ar}), 71.0 (s, 4C, C_{Ar}) ppm; MS (APCI-DIP) *m/z* (%): 213.97 (20) $[M - CHO]^+$, 242.97 (100) $[M + H^+]^+$, Anal. Calcd for $C_{12}H_{10}FeO_2$: C, 59.55; H, 4.16. Found: C, 59.74; H, 4.15.

1,1'-ferrocenedimethanol (5). In a 250 mL round-bottom flask, 1,1'-ferrocenedicarboxaldehyde (7.53 g, 31.1 mmol) was dissolved in a mixture of dry THF/MeOH (1:1, 160 mL). The solution was cooled in a water bath, and $NaBH_4$ tablets (9.42 g, 249 mmol) were added under vigorous stirring in four portions over a period of 2 h. Then, the mixture was stirred at RT overnight (20 h). To the orange solution, an aq. HCl solution (2.5M, 20 mL) was slowly added and stirred at RT for 5 min. The formed suspension was transferred to a separation-funnel, brine (100 mL) was added, and the phases were separated. The organic phase was washed with additional brine (100 mL). The combined aqueous phases were extracted with Et_2O (5 x 50 mL). The combined organic phases were dried over $MgSO_4$, filtered, and the solvent was evaporated giving a yellow-orange solid. Recrystallization from EtOAc/pentanes (200 mL; 3:1; hot filtration), and cooling in an ice-bath gave a first crop of crystals which were recovered via filtration and washed with -20 °C pentanes (2 x 30 mL). Storage of the filtrate at -20 °C gave a second crop of crystalline material. **5** was obtained in form of orange crystals (6.84 g, 27.8 mmol, 89%). ^{1}H NMR (400 MHz, $CDCl_3$): δ = 4.36 (s, 4H, CH_2), 4.27 (s, 4H, H_{Ar}), 4.22 (s, 4H, H_{Ar}), 3.32 (s_{br}, 2H, OH) ppm; $^{13}C\{^{1}H\}$ NMR (100 MHz, $CDCl_3$): δ = 84.8 (s, 2C, C_{Ar}), 69.1 (s, 4C, C_{Ar}), 68.2 (s, 4C, C_{Ar}), 60.5 (s, 2C, CH_2) ppm; MS (ESI+) *m/z* (%): 246.05 (40) $[M^+]^+$, 269.05 (100) $[M + Na^+]^+$, Anal. Calcd for $C_{12}H_{14}FeO_2$: C, 58.57; H, 5.73. Found: C, 58.83; H, 5.79.

1,1'-ferrocene-bis(diethylmethylphosphonate) (6). In a 50 mL Schlenk flask, 1,1'-ferrocenedimethanol (2.26 g, 10.0 mmol), tetrabutylammonium iodide (148 mg, 400 µmol) and $P(OEt)_3$ (5.14 mL, 30.0 mmol) were combined, and the mixture was heated at 125 °C for 24 h. The mixture was cooled to RT and excess of $P(OEt)_3$ was removed in vacuo. Flash chromatography (Silica; 2CV neat EtOAc, then gradient to neat EtOH over 14 CV) gave **6** as a brown viscous oil (2.34 g, 4.80 mmol 48%). ^{1}H NMR (400 MHz, $CDCl_3$): δ = 4.15 (s, 4H, H_{Ar}), 4.07–4.03 (m, 4H, H_{Ar}), 4.02–3.93 (m, 8H, CH_2CH_3), 2.85 (d, $^{2}J_{PH}$ = 19.5 Hz, 4H, CH_2), 1.24 (t, $^{3}J_{HH}$ = 7.0 Hz, 12H, CH_2CH_3) ppm; $^{13}C\{^{1}H\}$ NMR (100 MHz, $CDCl_3$): δ = 78.3 (d, 2C, $^{2}J_{PC}$ = 3.4 Hz, C_{Ar}), 70.2 (d, 4C, $^{3}J_{PC}$ = 3.0 Hz, C_{Ar}), 69.0 (s, 4C, C_{Ar}), 62.1 (d, 4C, $^{2}J_{PC}$ = 6.7 Hz, CH_2CH_3), 27.8 (d, 2C, $^{1}J_{PC}$ = 139 Hz, CH_2), 16.4 (d, 4C, $^{3}J_{PC}$ = 5.9 Hz, CH_2CH_3) ppm; $^{31}P\{^{1}H\}$ NMR (202 MHz, $CDCl_3$): δ = 25.0 (s) ppm; IR (ATR) $\tilde{\nu}$ = 1232 (P=O), 1097 (P–OEt) cm^{-1}; MS (ESI+) *m/z* (%): 509.08 (100) $[M + Na^+]^+$, 995.21

(65) [2M + Na^+]$^+$; Anal. Calcd for $C_{20}H_{32}FeO_6P_2$: C, 49.40; H, 6.63. Found: C, 46.71; H, 6.94. Deviation due to a contamination with DCM.

1,1'-ferrocene-bis(methylphosphonic acid) (7). 1,1'-ferrocenylene-bis(diethylmethylphosphonate) (4.86 g, 10.0 mmol) was dissolved in dry DCM (100 mL) and Me_3SiBr (10.6 mL, 80.0 mmol) was drop wise added via syringe at RT. The mixture was stirred at RT overnight (16 h). H_2O (1.44 mL, 80 mmol) was added, and the mixture was vigorously stirred for 15 min. The black aqueous phase was separated and the solvent from the organic phase was removed. The yellow-greenish residue was dissolved in aq. NaOH solution (1.0M, 100 mL), and an excess of sodium metabisulfite was added under stirring to reduce the solution to a yellow color. The mixture was acidified with conc. HCl resulting in the precipitation of a solid. The precipitate was recovered via filtration over a Büchner-funnel with suction, washed with a mixture of EtOAc/THF (2:1; 2 x 50 mL), and air dried. Compound **7** was obtained as a dark yellow to ocher solid (2.96 g, 7.92 mmol, 79%). ^1H NMR (400 MHz, DMSO-d_6): δ = 4.32 (s_{vbr}, 4H, OH), 4.09 (s, 4H, H_{Ar}), 4.01 (s, 4H, H_{Ar}), 2.66 (d, $^2J_{PH}$ = 19.3 Hz, 4H, CH_2) ppm; $^{13}C\{^1H\}$ NMR (100 MHz, DMSO-d_6): δ = 80.4 (d, 2C, $^2J_{PC}$ = 2.6 Hz, C_{Ar}), 70.2 (d, 4C, $^3J_{PC}$ = 2.7 Hz, C_{Ar}), 67.9 (s, 4C, C_{Ar}), 29.3 (d, 2C, $^1J_{PC}$ = 134 Hz, CH_2) ppm; $^{31}P\{^1H\}$ NMR (202 MHz, DMSO-d_6): δ = 20.9 (s) ppm; MS (ESI+) *m/z* (%): 396.96 (100) [M + Na^+]$^+$, 770.97 (10) [2M + Na^+]$^+$, Anal. Calcd for $C_{12}H_{16}FeO_6P_2$: C, 38.53; H, 4.31. Found: C, 38.72; H, 4.40.

Sodium 1,1'-ferrocene-bis(methylphosphonate) (8). 1,1'-ferrocenylene-bis(methyl-phosphonic acid) (5.51 g, 6.71 mmol) was dissolved in a mixture of EtOH (300 mL), THF (300 mL), and DMSO (70 mL) in a 1 L round-bottom flask. Then, NaOH beads (1.08 g, 27.0 mmol) were added in one portion and the greenish mixture was vigorously stirred at RT for 45 min. The formed precipitate was recovered via filtration over a pleated filter, washed with EtOH (2 x 30 mL), and air-dried. Compound **8** was obtained as a beige solid (2.54 g, 5.50 mmol, 37%). ^1H NMR (400 MHz, DMSO-d_6): δ = 4.11 (s_{br}, 4H, H_{Ar}), 3.99 (s, 4H, H_{Ar}), 3.64 (s_{vbr}, 4H, OH), 2.56 (s_{br}, 4H, CH_2) ppm; $^{13}C\{^1H\}$ NMR (100 MHz, DMSO-d_6): δ = 82.3 (d, 2C, $^2J_{PC}$ = 2.8 Hz, C_{Ar}), 69.7 (d, 4C, $^3J_{PC}$ = 4.6 Hz, C_{Ar}), 67.0 (s, 4C, C_{Ar}), 30.1 (d, 2C, $^1J_{PC}$ = 132 Hz, CH_2) ppm; $^{31}P\{^1H\}$ NMR (202 MHz, D_2O): δ = 18.8 (s) ppm; IR (ATR) $\tilde{\nu}$ = 1238 (P=O), 1105 (P–ONa) cm^{-1}; MS (ESI+) *m/z* (%): 440.93 (100) [M - Na^+ 2H^+]$^+$, 462.91 (80) [M + H^+]$^+$, 484.89 (20) [M + Na^+]$^+$; Anal. Calcd for $C_{12}H_{12}FeNa_4O_6P_2$: C, 31.20; H, 2.62. Found: C, 28.63; H, 3.87. Deviation due to a contamination with EtOH.

2. NMR Spectra

1,1'-Ferrocene-bis(diethyl phosphonate) (**1**)

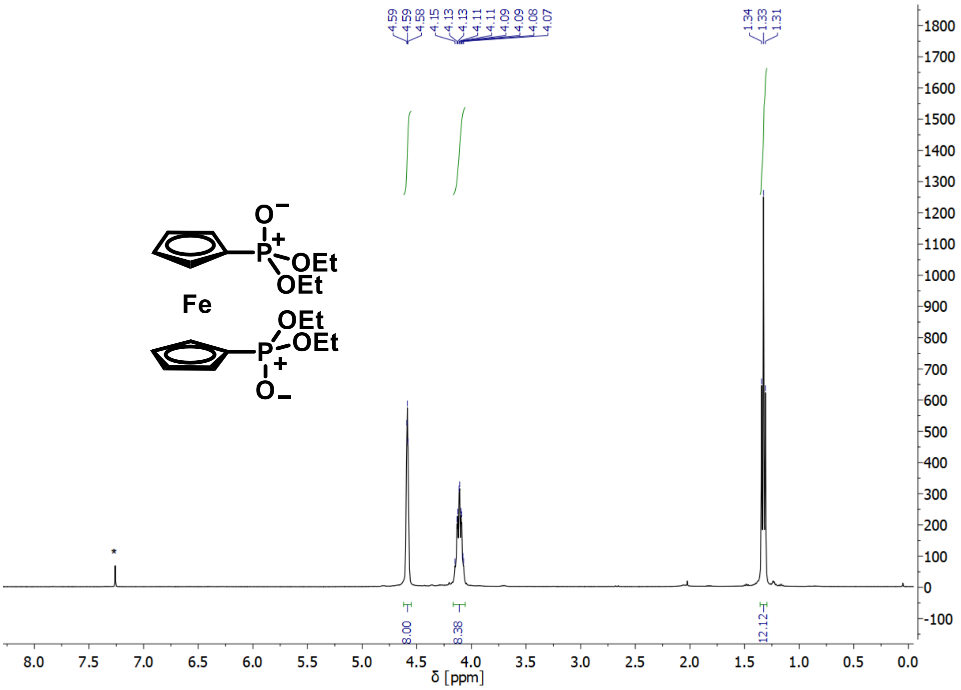

Figure S1. ^{1}H NMR spectrum of 1,1'-ferrocene-bis(diethyl phosphonate) (**1**) in $CDCl_3$ (*).

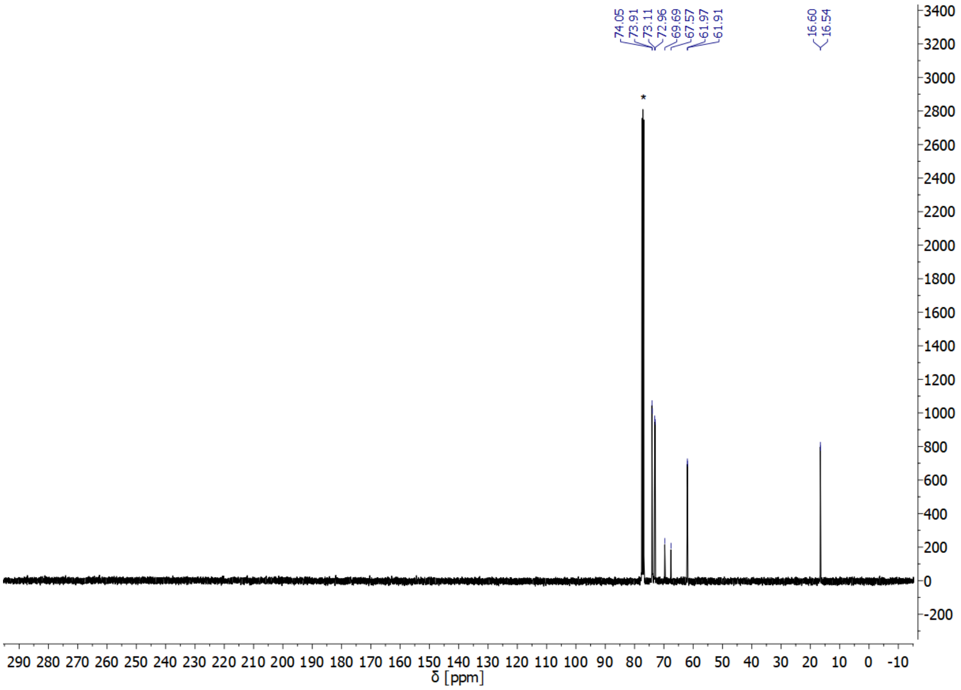

Figure S2. ^{13}C NMR spectrum of 1,1'-ferrocene-bis(diethyl phosphonate) (**1**) in $CDCl_3$ (*).

1,1'-Ferrocene-bis(phosphonic acid) (**2**)

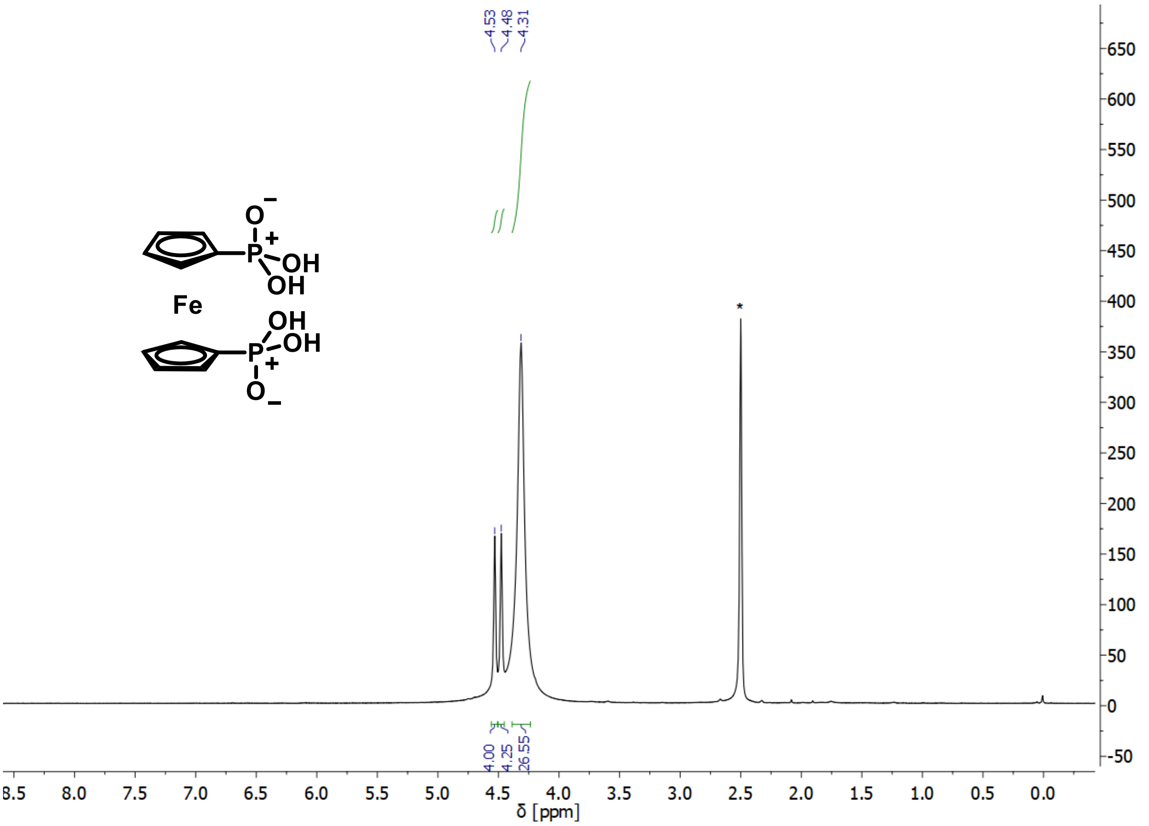

Figure S3. ^{1}H NMR spectrum of 1,1'-ferrocene-bis(phosphonic acid) (**2**) in DMSO-d_6 (*).

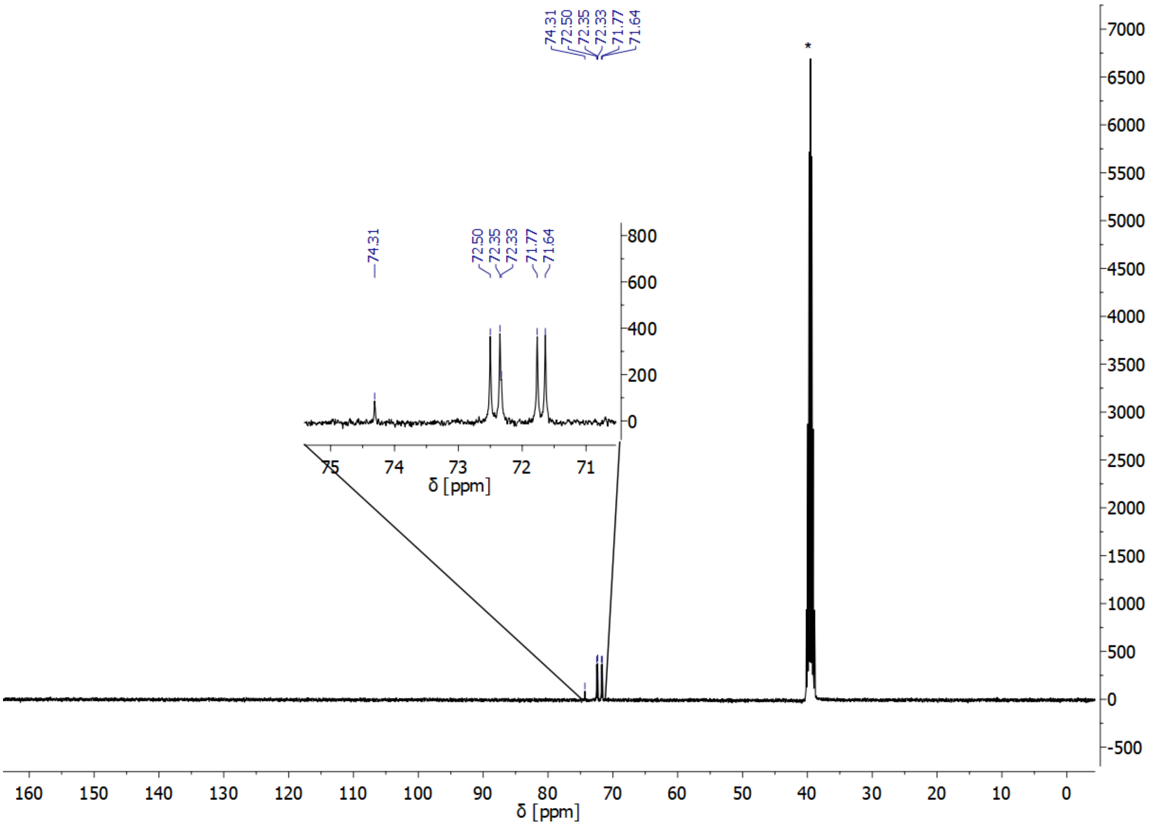

Figure S4. ^{13}C NMR spectrum of 1,1'-ferrocene-bis(phosphonic acid) (**2**) in DMSO-d_6 (*).

Sodium 1,1'-ferrocene-bis(phosphonate) (**3**)

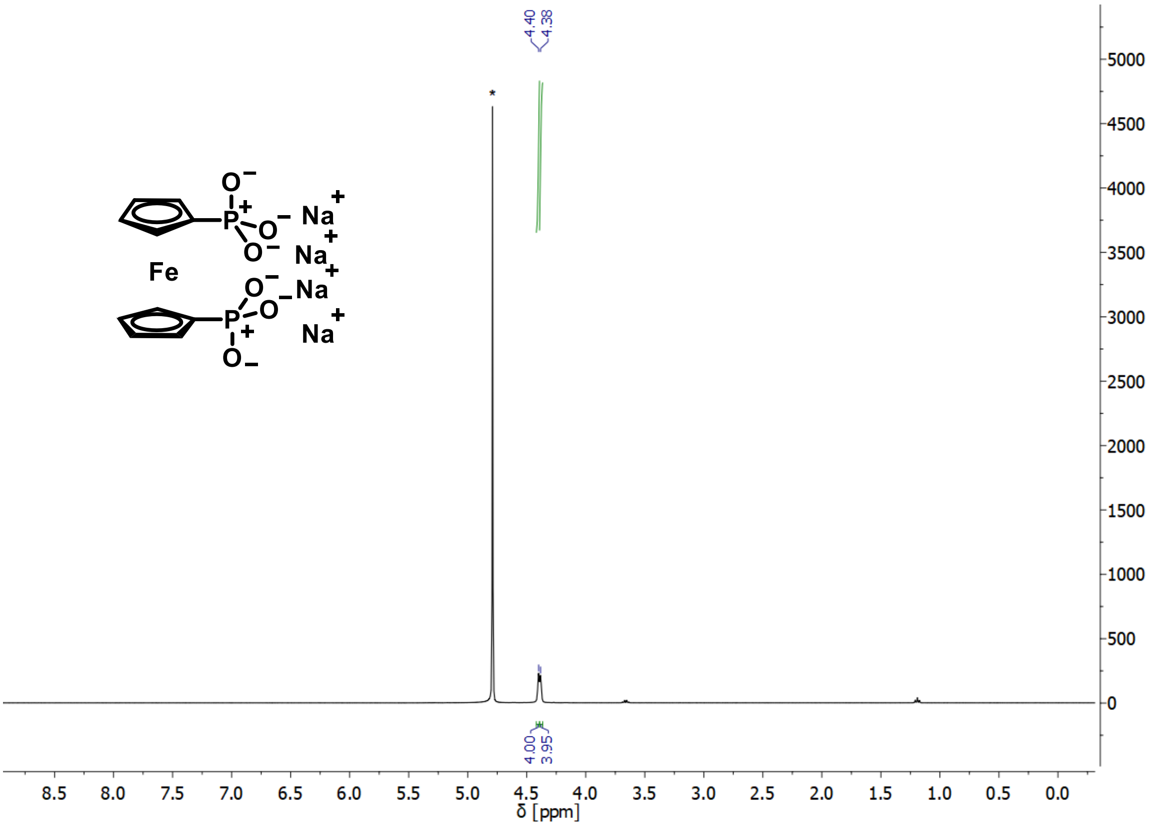

Figure S5. ^{1}H NMR spectrum of sodium 1,1'-ferrocene-bis(phosphonate) (**3**) in D_2O (*).

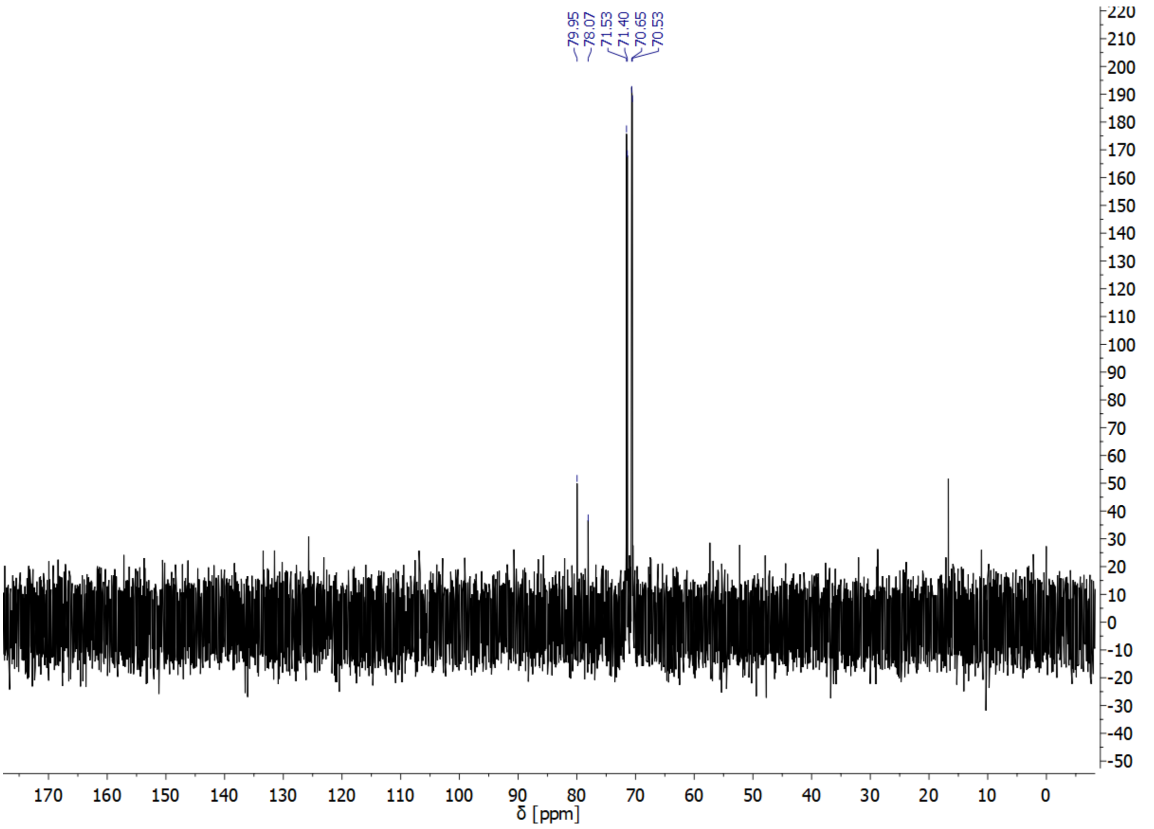

Figure S6. ^{13}C NMR spectrum of sodium 1,1'-ferrocene-bis(phosphonate) (**3**) in D_2O.

1,1'-Ferrocenedicarboxaldehyde (**4**)

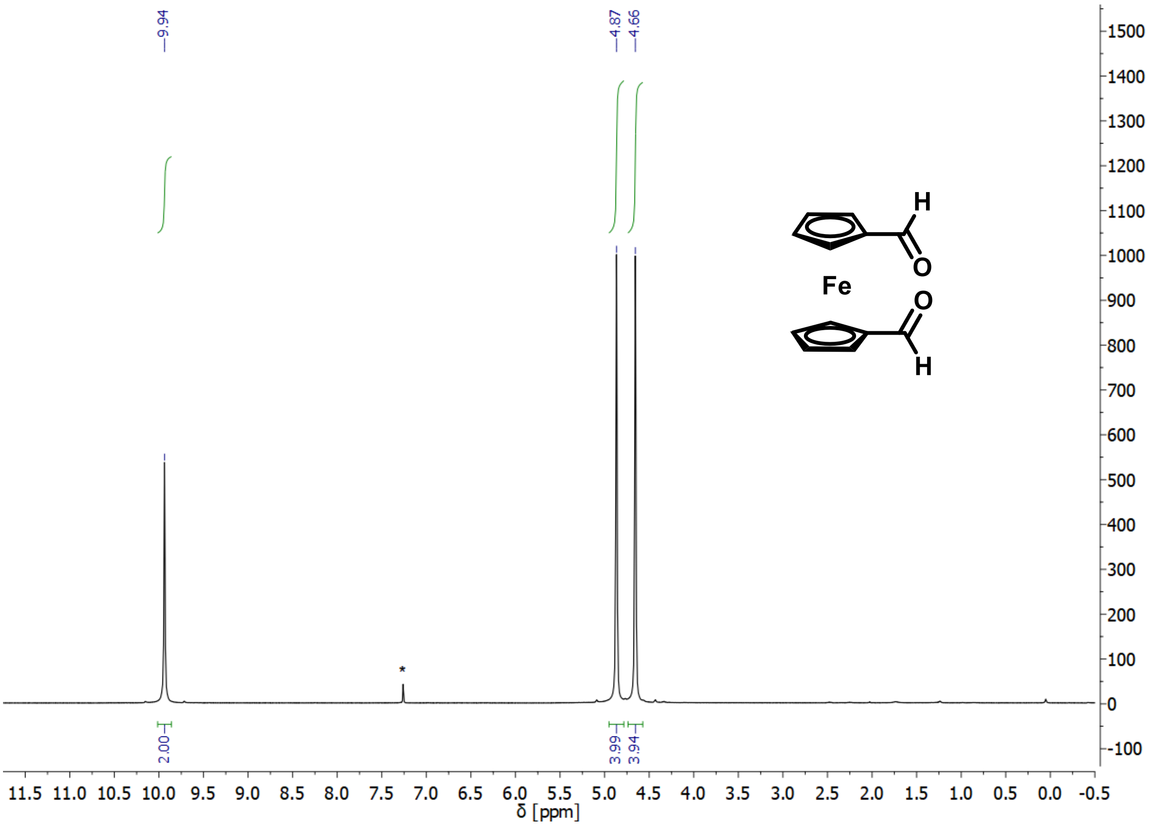

Figure S7. ^{1}H NMR spectrum of 1,1'-ferrocenedicarboxaldehyde (**4**) in $CDCl_3$ (*).

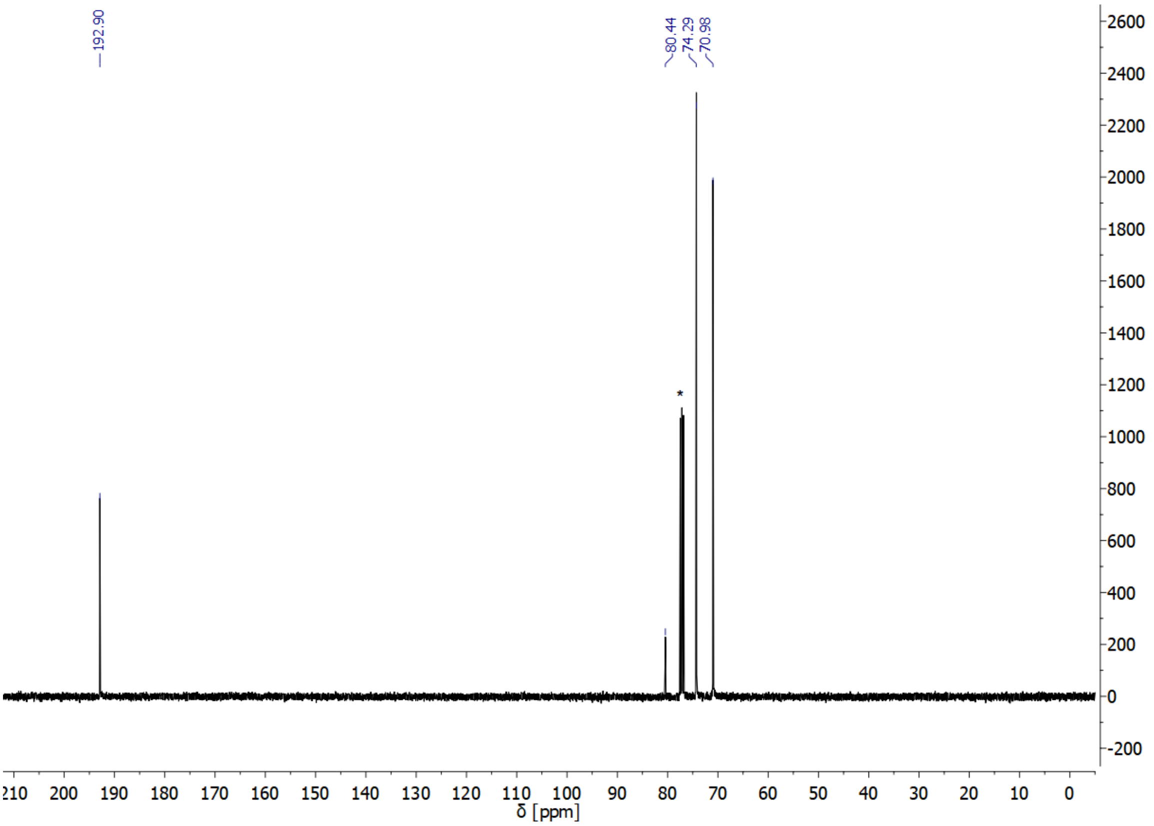

Figure S8. ^{13}C NMR spectrum of 1,1'-ferrocenedicarboxaldehyde (**4**) in $CDCl_3$ (*).

1,1'-Ferrocenedimethanol (5)

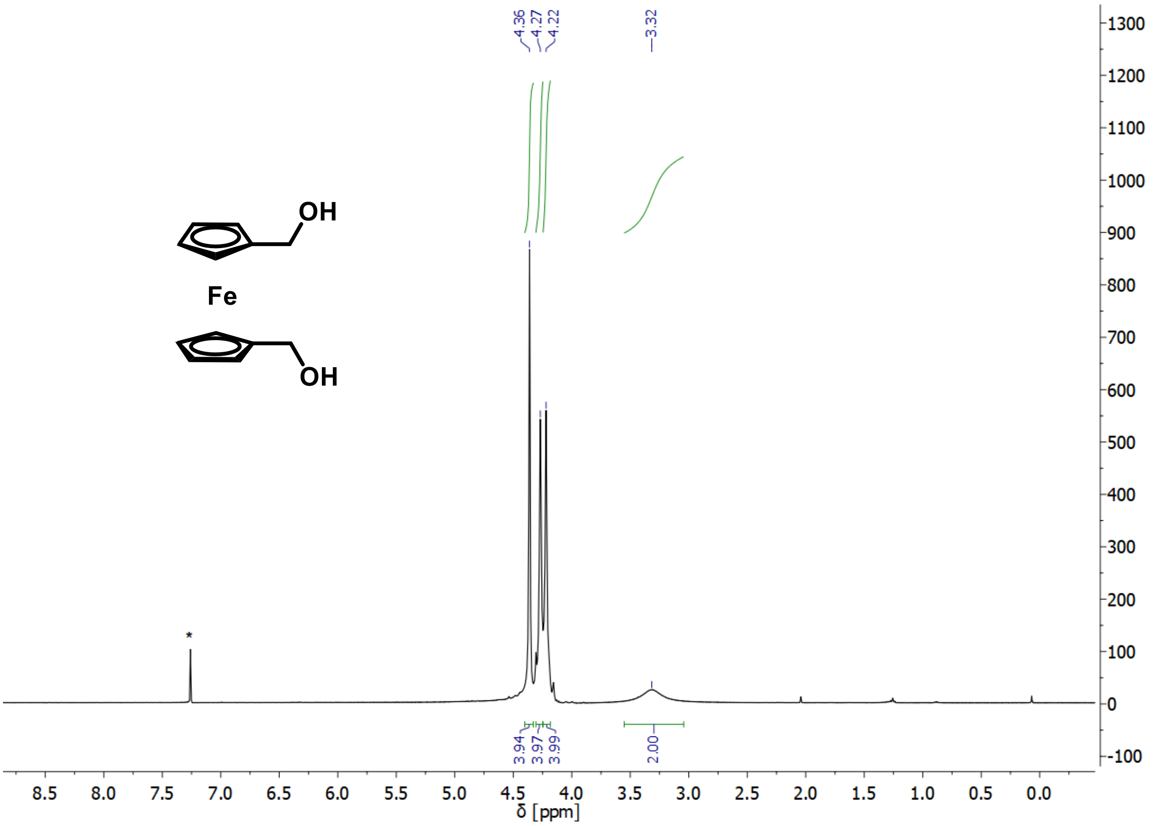

Figure S9. ^{1}H NMR spectrum of 1,1'-ferrocenedimethanol (**5**) in $CDCl_3$ (*).

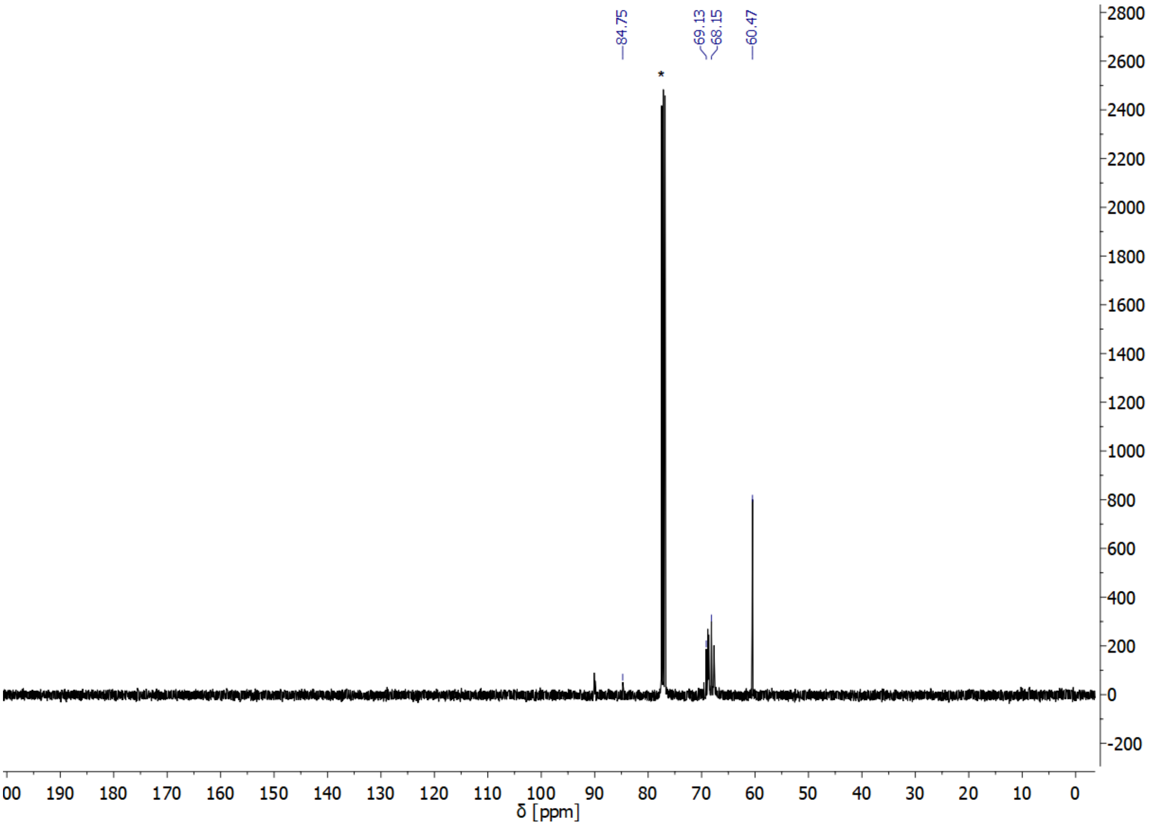

Figure S10. ^{13}C NMR spectrum of 1,1'-ferrocenedimethanol (**5**) in $CDCl_3$ (*).

1,1'-Ferrocene-bis(diethylmethylphosphonate) (**6**)

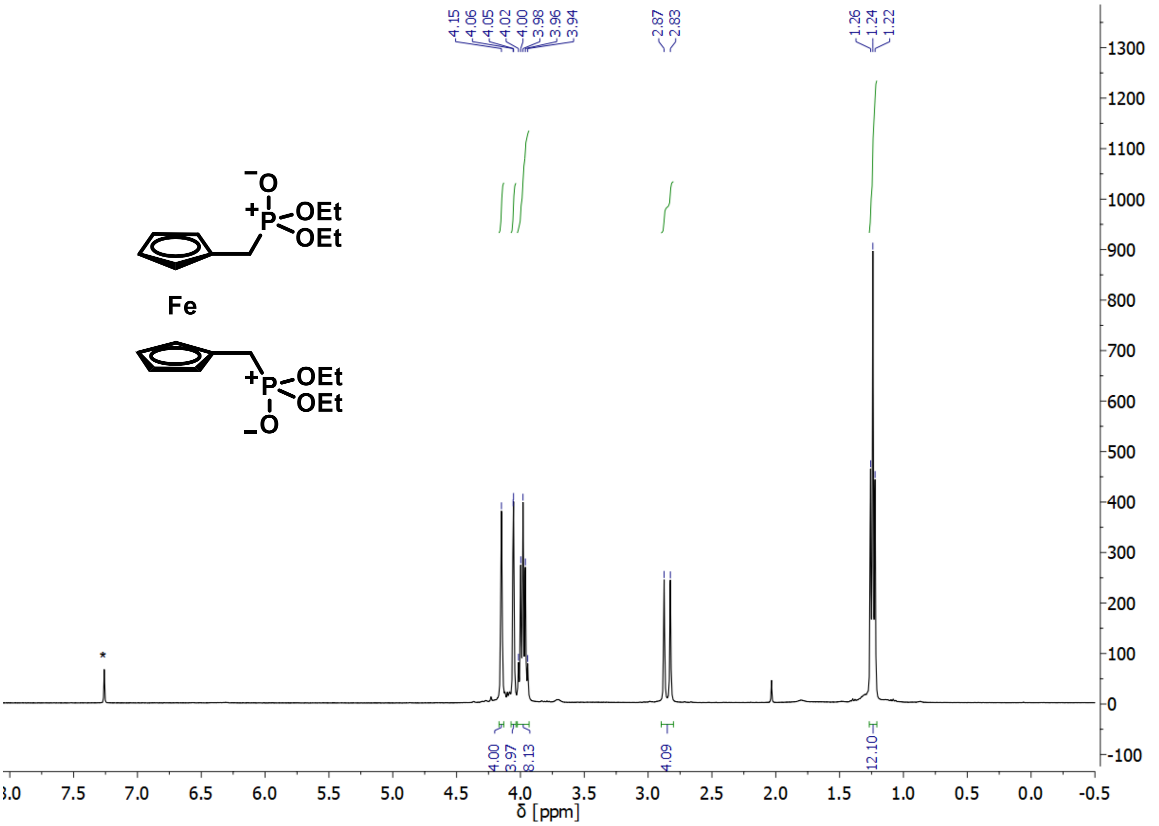

Figure S11. ^{1}H NMR spectrum of 1,1'-ferrocene-bis(diethylmethylphosphonate) (**6**) in $CDCl_3$ (*).

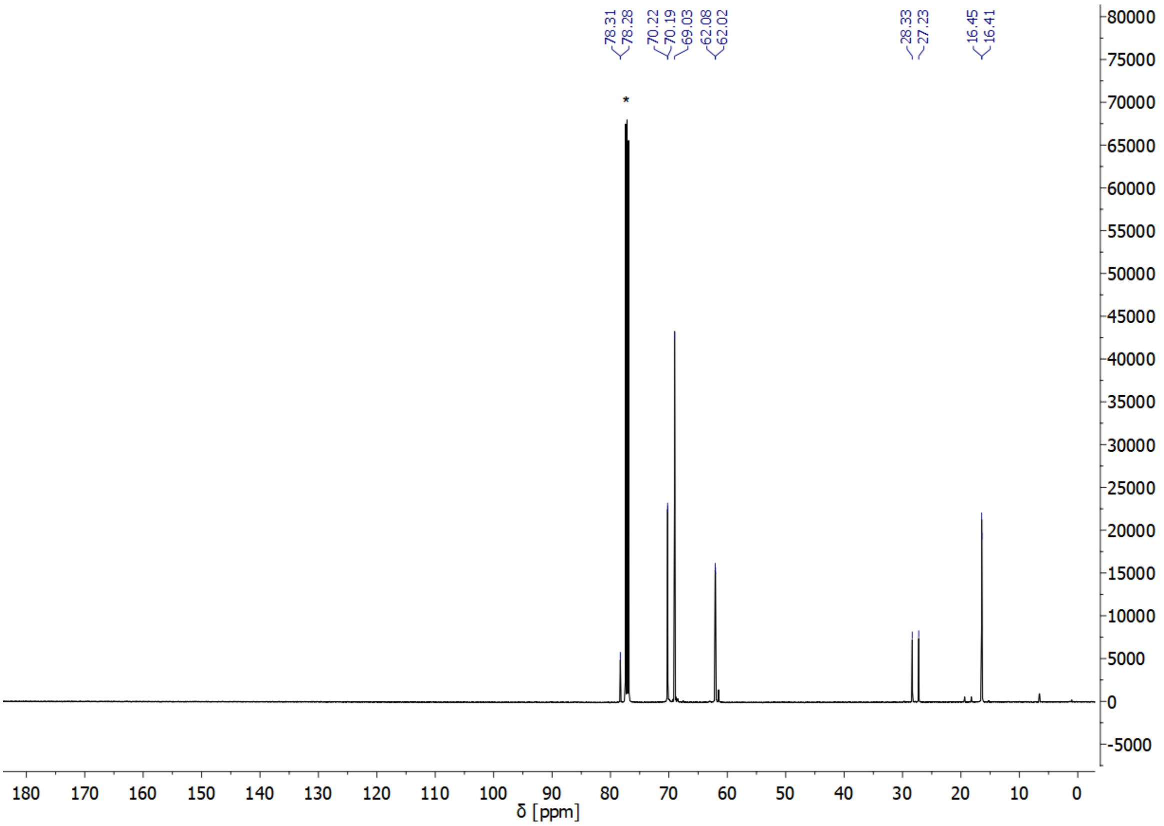

Figure S12. $^{13}C\{^{1}H\}$ NMR spectrum of 1,1'-ferrocene-bis(diethylmethylphosphonate) (**6**) in $CDCl_3$ (*).

1,1'-Ferrocene-bis(methylphosphonic acid) (**7**)

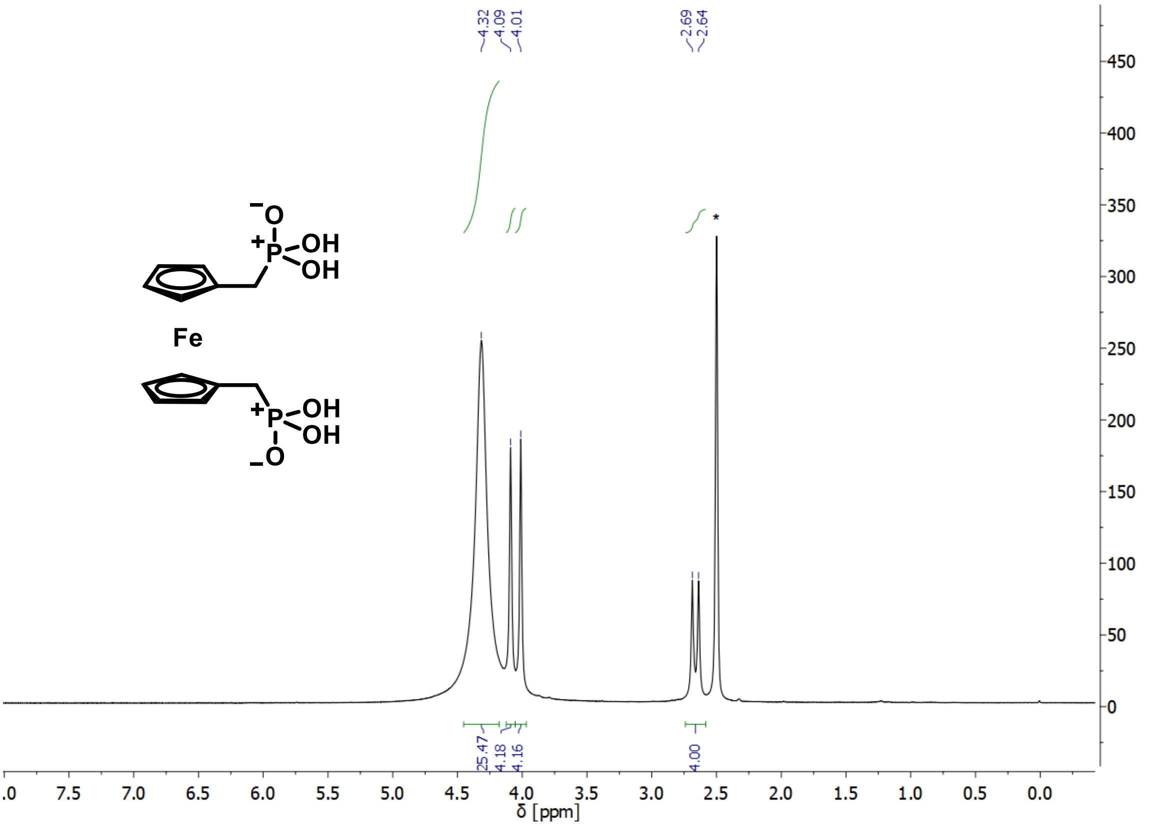

Figure S13. ^{1}H NMR spectrum of 1,1’-ferrocene-bis(methylphosphonic acid) (**7**) in DMSO-d_6 (*).

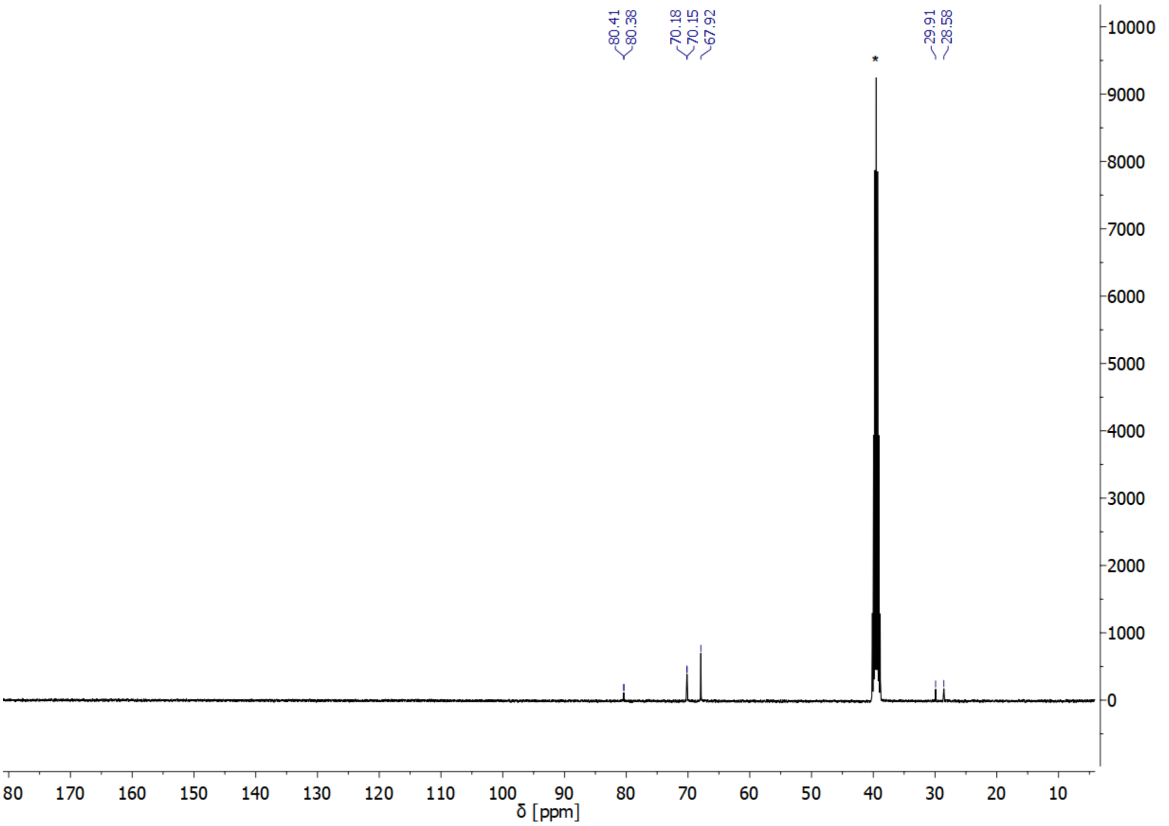

Figure S14. $^{13}C\{^{1}H\}$ NMR spectrum of 1,1’-ferrocene-bis(methylphosphonic acid) (**7**) in DMSO-d_6 (*).

Sodium 1,1'-ferrocene-bis(methylphosphonate) (8)

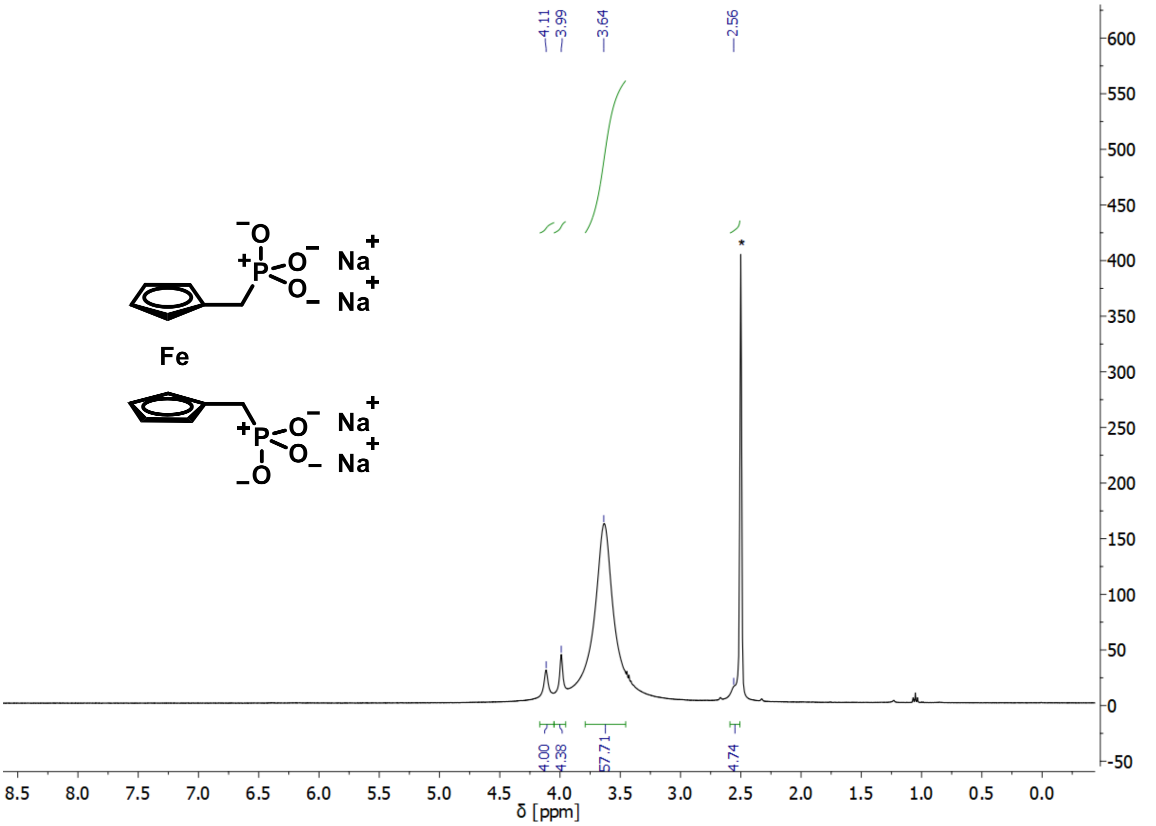

Figure S15. ^{1}H NMR spectrum of sodium 1,1'-ferrocene-bis(methylphosphonate) (**8**) in DMSO-d$_6$ (*).

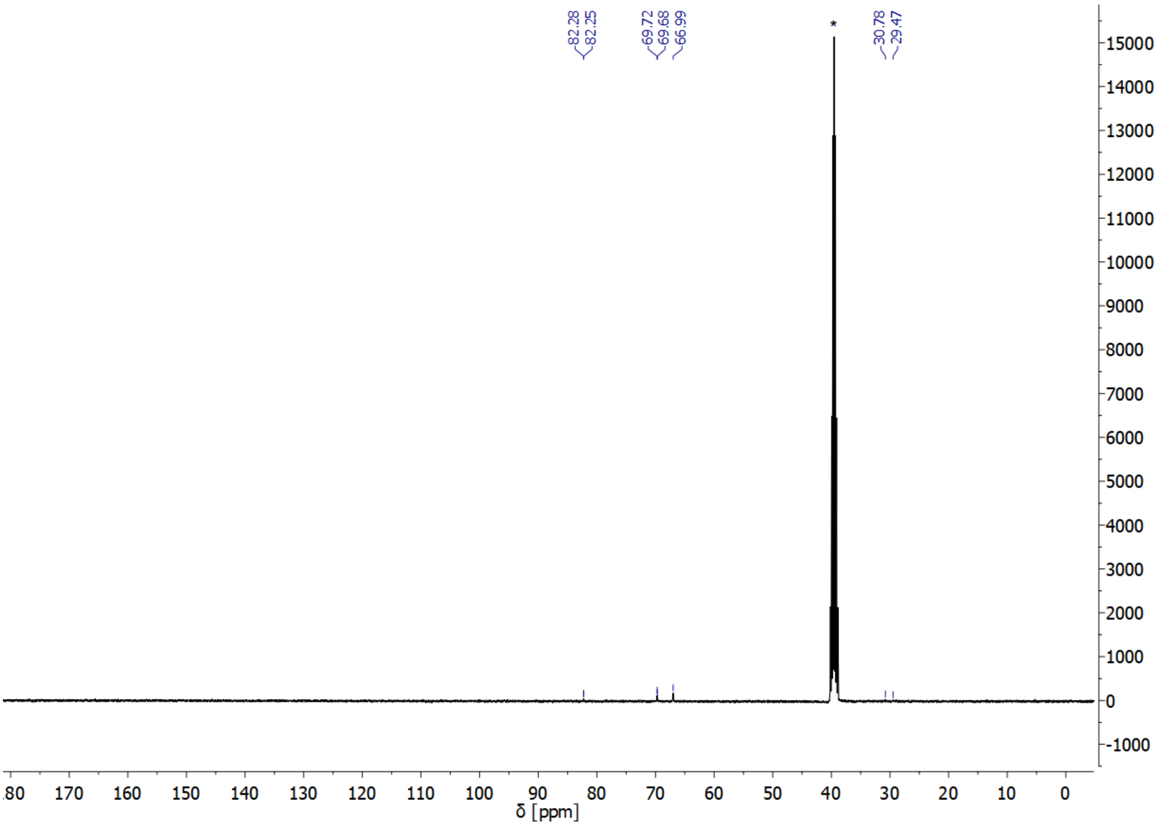

Figure S16. ^{13}C{^{1}H} NMR spectrum of sodium 1,1'-ferrocene-bis(methylphosphonate) (**8**) in DMSO-d$_6$ (*).

3. ^{31}P NMR Investigations of Ageing Solutions of 3 & 8 at Different pH values under Air & Argon

Table S1. Ageing experiments of compound **3** in 0.5M H_3PO_4, phosphate buffer and 1M NaOH **under air** over 21 days.

Day	Precipitate?	in 0.5M H_3PO_4	Precipitate?	In phosphate buffer	Precipitate?	in 1M NaOH
21	No	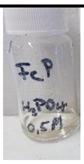	Slight (pale-yellow)	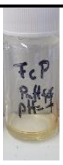	Medium (reddish orange)	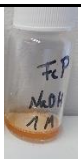
14	No	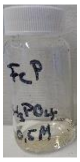	Slight (pale-yellow)	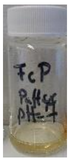	Medium (reddish orange)	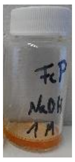
7	No	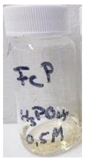	No	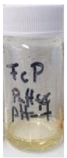	Medium (reddish orange)	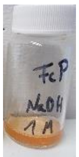
4	No	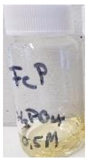	No	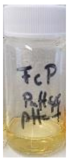	Medium (reddish orange)	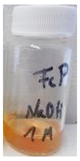
2	No	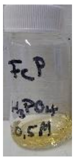	No	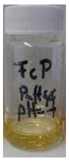	Slight (reddish orange)	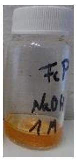
1	No	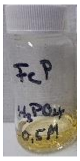	No	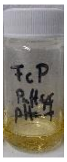	No	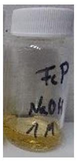

Table S2. Ageing experiments of compound **8** in 0.5M H_3PO_4, phosphate buffer and 1M NaOH **under air** over 21 days.

Day	Precipitate?	in 0.5M H_3PO_4	Precipitate?	In phosphate buffer	Precipitate?	in 1M NaOH
21	Medium (brownish)	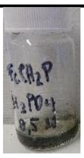	Slight (pale-yellow)	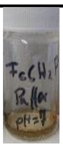	Medium (reddish orange)	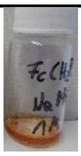
14	Medium (brownish)	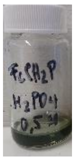	Slight (pale-yellow)	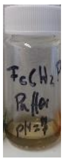	Medium (reddish orange)	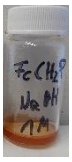

7	Medium (brownish)	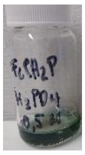	Slight (pale-yellow)	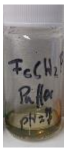	Medium (reddish orange)	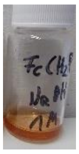
4	Medium (brownish)	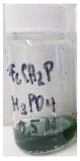	No	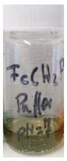	Medium (reddish orange)	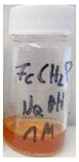
2	Slight (brownish)	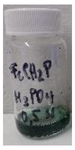	No	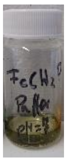	Slight (reddish orange)	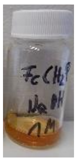
1	No	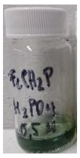	No	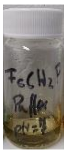	No	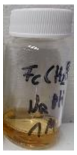

Table S3. Ageing experiments of compound **3** in 0.5M H3PO4, phosphate buffer and 1M NaOH **under argon** over 21 days.

Day	Precipitate?	in 0.5M H_3PO_4	Precipitate?	In phosphate buffer	Precipitate?	in 1M NaOH
21	Slight (pale-yellow)	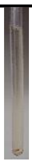	Slight (yellow brownish)	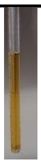	Medium (brownish orange)	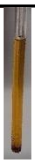
14	Slight (pale-yellow)	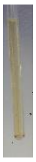	Slight (pale-yellow)	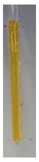	Medium (green brownish)	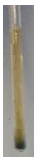
7	Slight (pale-yellow)	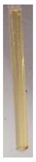	Slight (pale-yellow)	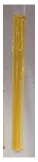	Medium (green brownish)	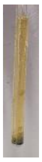
4	No	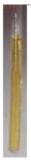	No	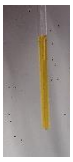	Slight (green brownish)	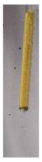
2	No	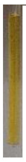	No	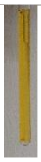	Slight (green brownish)	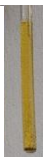

1	No	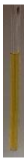	No	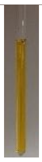	No	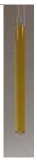

Table S4. Ageing experiments of compound **8** in 0.5M H_3PO_4, phosphate buffer and 1M NaOH **under argon** over 21 days.

Day	Precipitate?	in 0.5M H_3PO_4	Precipitate?	In phosphate buffer	Precipitate?	in 1M NaOH
21	Strong (brownish)	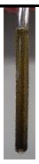	Slight (pale-yellow)	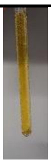	Strong (brownish orange)	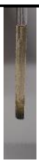
14	Strong (green-brownish)	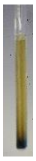	Slight (pale-yellow)	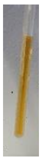	Medium (reddish orange)	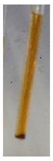
7	Strong (green-brownish)	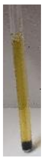	Slight (pale-yellow)	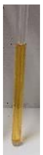	Medium (reddish orange)	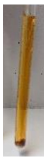
4	Strong (dark green)	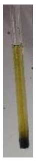	Slight (pale-yellow)	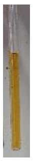	Medium (reddish orange)	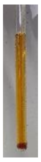
2	Strong (dark green)	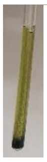	Slight (pale-yellow)	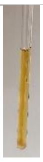	Slight (reddish orange)	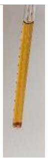
1	Strong (dark green)	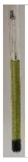	Slight (pale-yellow)	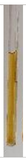	Slight (reddish orange)	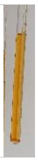

4. Stacked $^{31}P\{^{1}H\}$ NMR Spectra of Ageing Solutions of 3 & 8 at Different pH Values

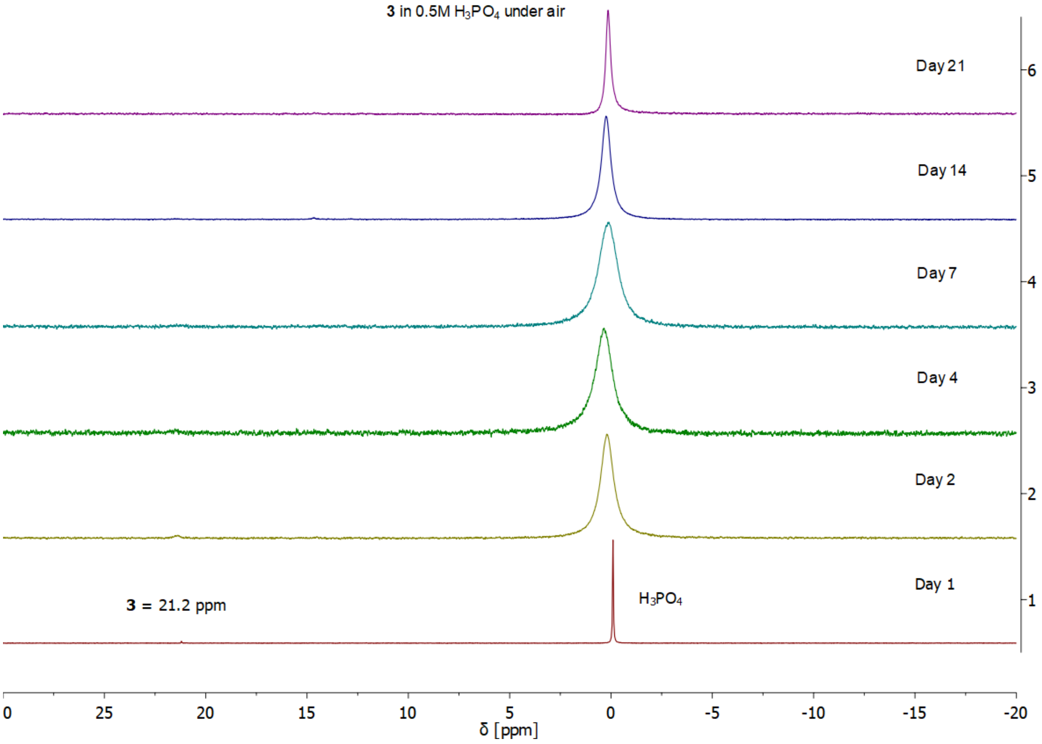

Figure S17. $^{31}P\{^{1}H\}$ NMR spectra of **3** in 0.5M H_3PO_4 under air at day 1, 2, 4, 7 ,14 and 21. Line broadening due to the formation of paramagnetic Fe(II) HS and/ or Fe(III) LS species.

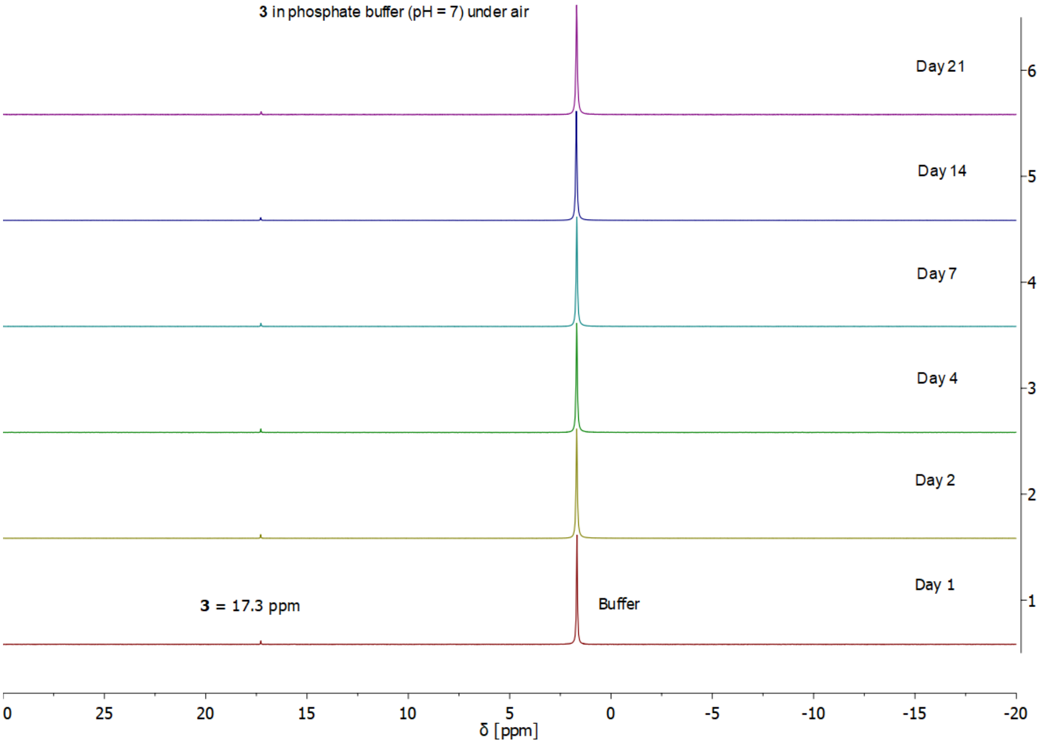

Figure S18. $^{31}P\{^{1}H\}$ NMR spectra of **3** in a phosphate buffer under air at day 1, 2, 4, 7 ,14 and 21.

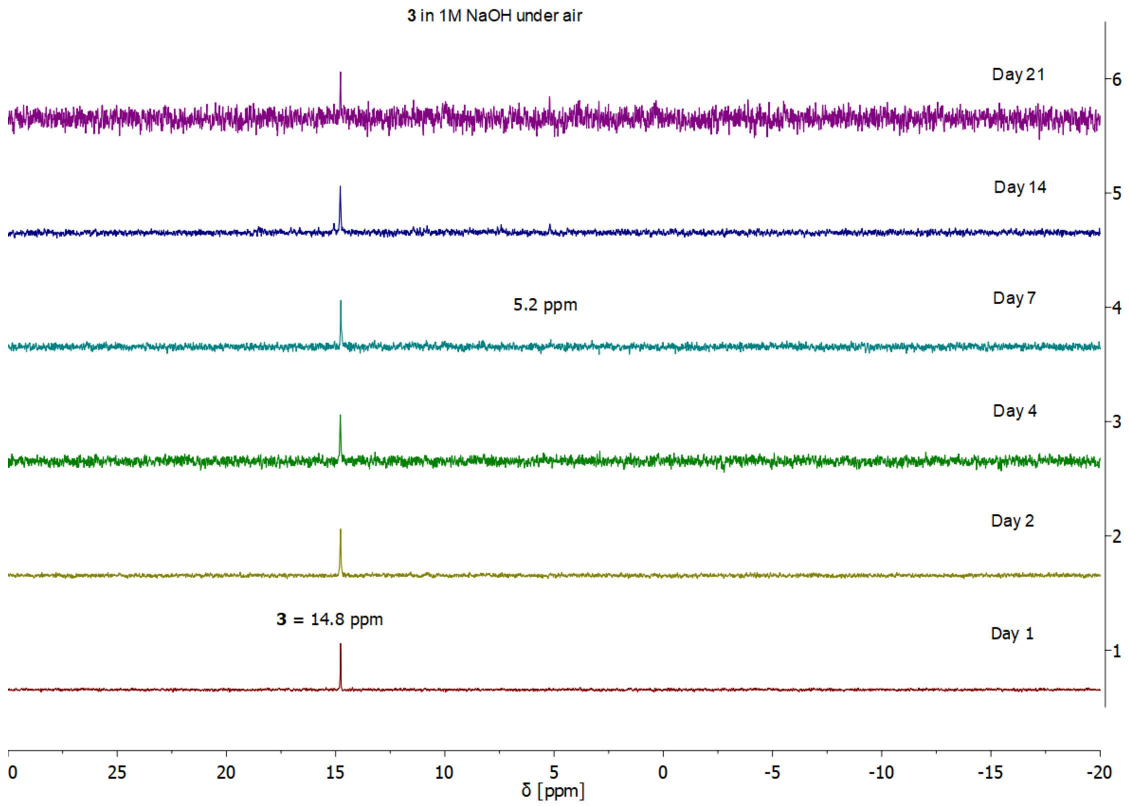

Figure S19. $^{31}P\{^{1}H\}$ NMR spectra of **3** in 1M NaOH under air at day 1, 2, 4, 7 ,14 and 21. The resonance emerging at 5.2 ppm can be assigned to a Na_2HPO_4 decomposition product.

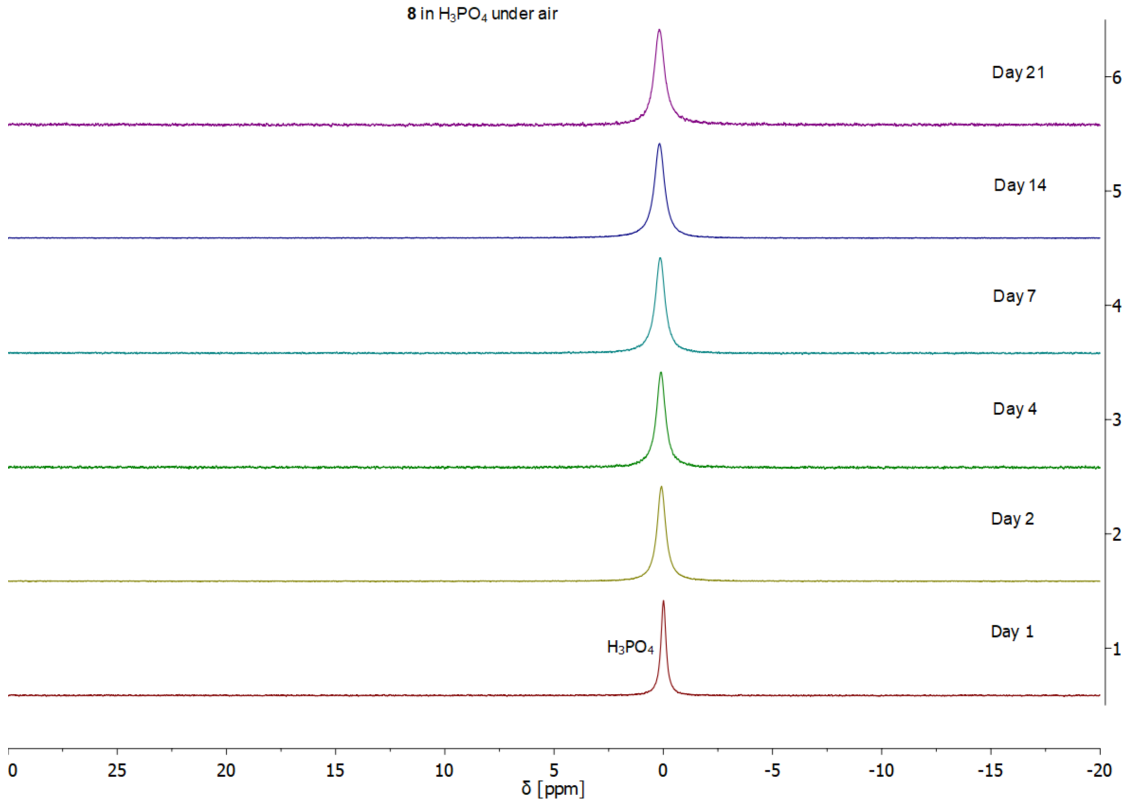

Figure S20. $^{31}P\{^{1}H\}$ NMR spectra of **8** in 0.5M H_3PO_4 under air at day 1, 2, 4, 7 ,14 and 21. Owing to the low concentration, no resonance of **8** was detected. Line broadening due to the formation of paramagnetic Fe(II) HS and/ or Fe(III) LS species.

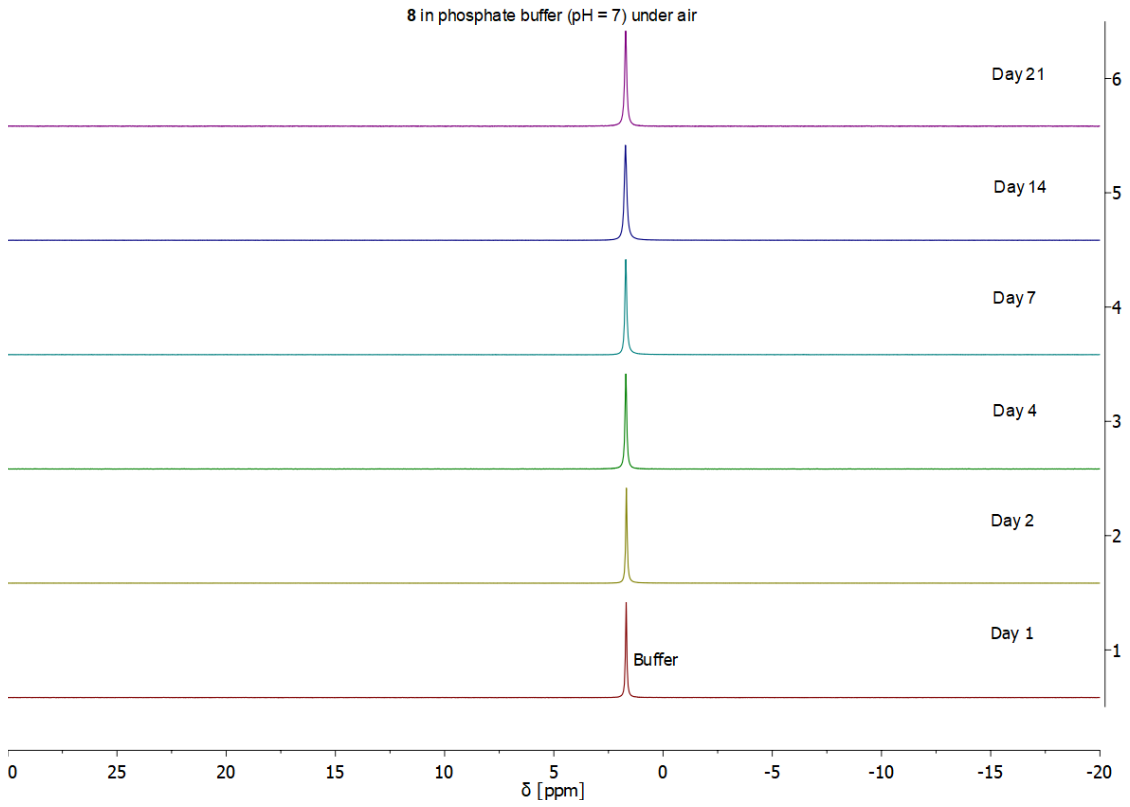

Figure S21. $^{31}P\{^{1}H\}$ NMR spectra of **8** in a phosphate buffer under air at day 1, 2, 4, 7 ,14 and 21. Owing to the low concentration, no resonance of **8** was detected.

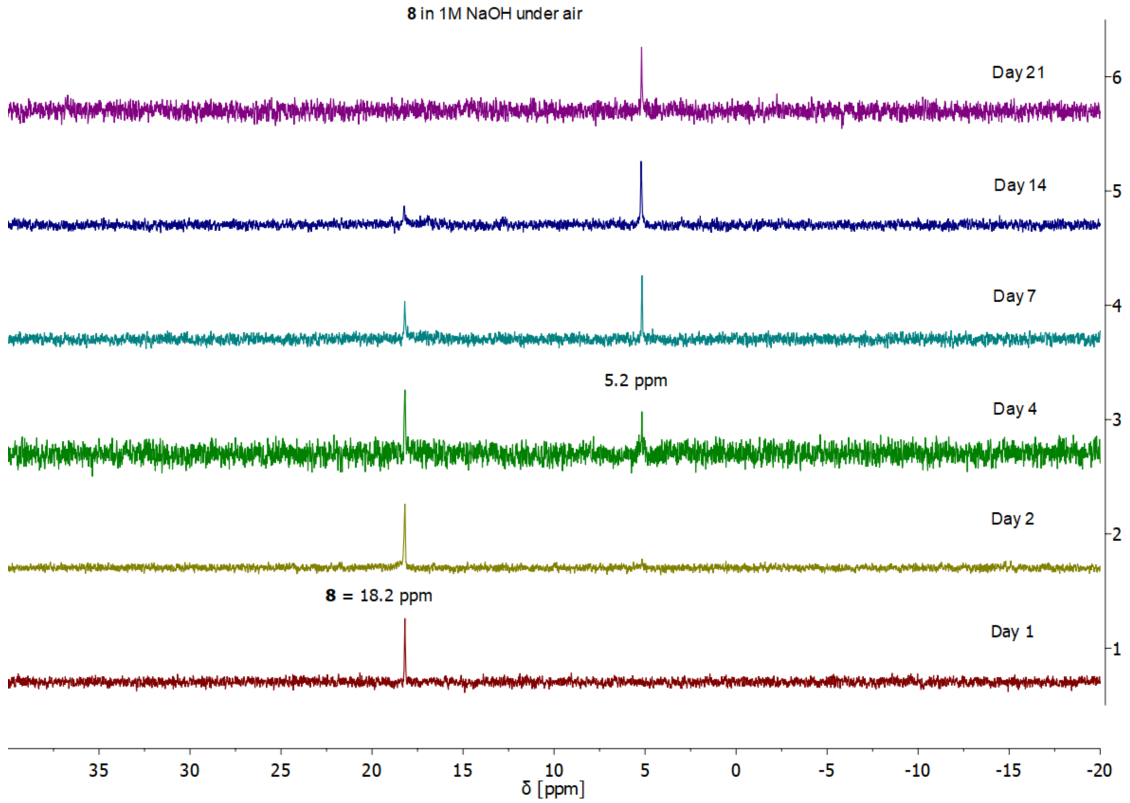

Figure S22. $^{31}P\{^{1}H\}$ NMR spectra of **8** in 1M NaOH under air at day 1, 2, 4, 7 ,14 and 21. The resonance emerging at 5.2 ppm can be assigned to a Na_2HPO_4 decomposition product.

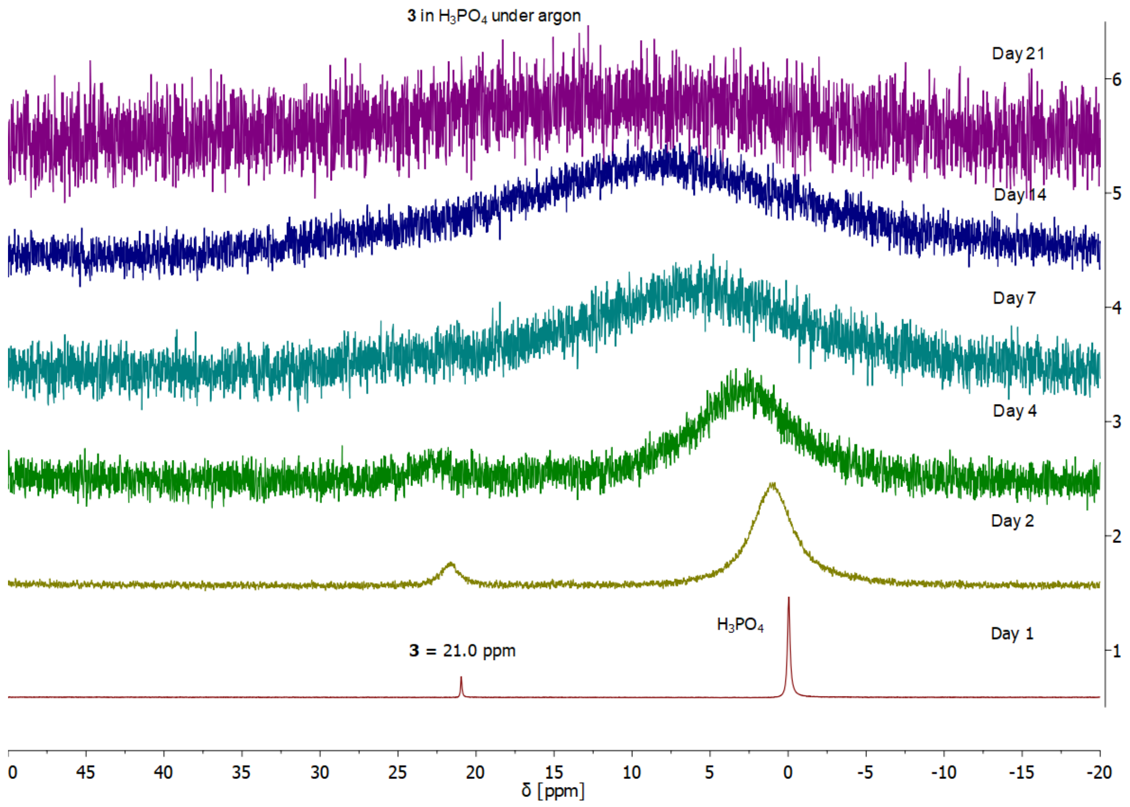

Figure S23. $^{31}P\{^{1}H\}$ NMR spectra of **3** in 0.5M H_3PO_4 under argon at day 1, 2, 4, 7 ,14 and 21. Pronounced line broadening due to the formation of strongly paramagnetic Fe(II) HS species.

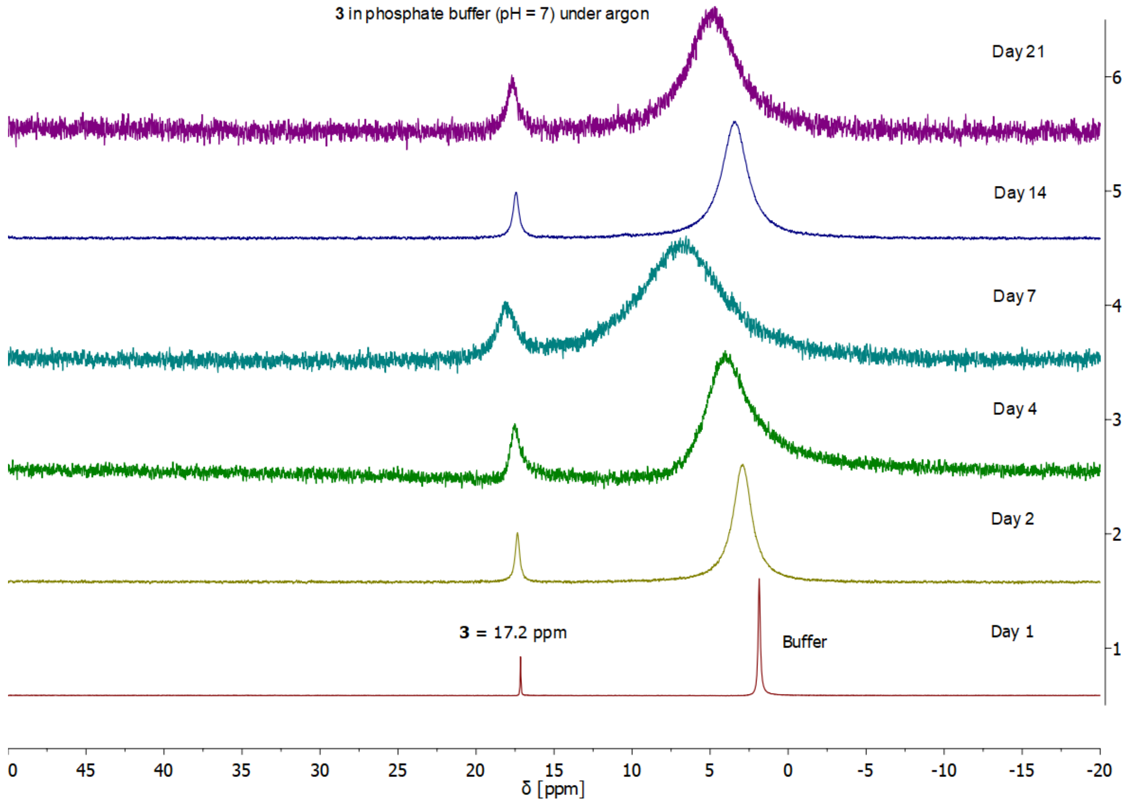

Figure S24. $^{31}P\{^{1}H\}$ NMR spectra of **3** in a phosphate buffer under argon at day 1, 2, 4, 7 ,14 and 21. Pronounced line broadening due to the formation of strongly paramagnetic Fe(II) HS species.

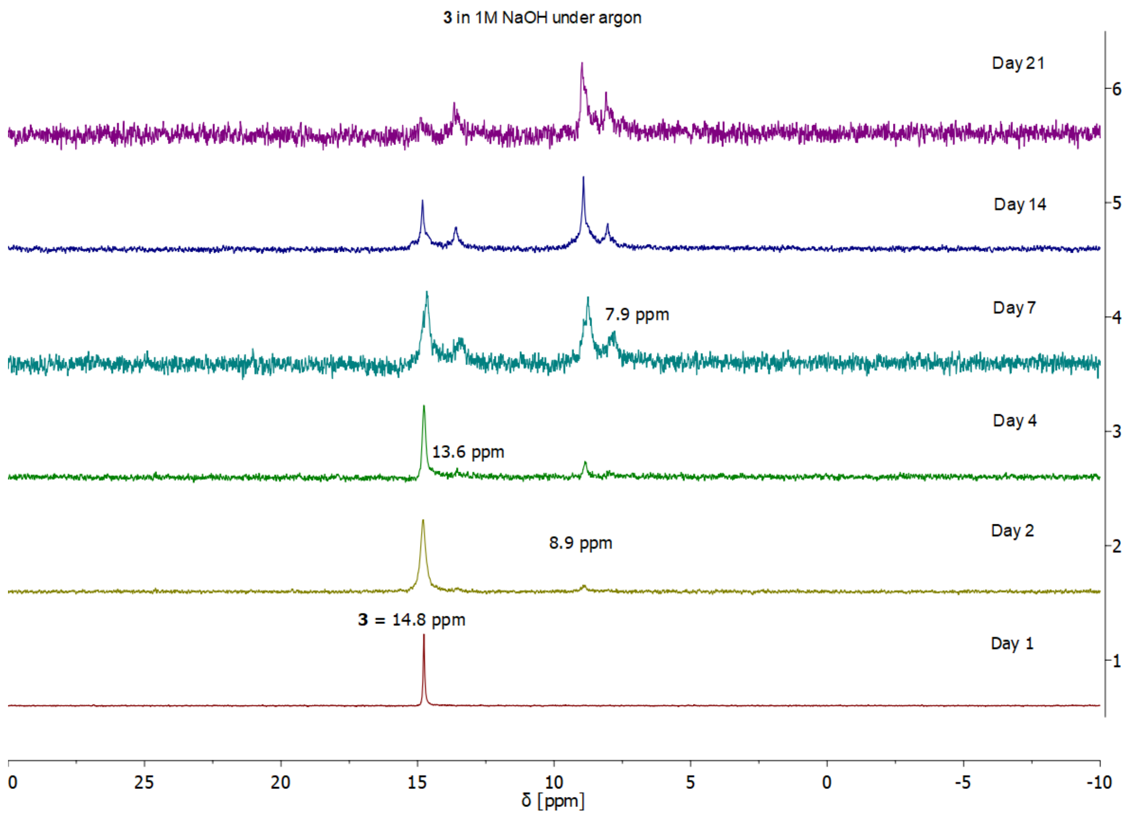

Figure S25. $^{31}P\{^{1}H\}$ NMR spectra of **3** in 1M NaOH under argon at day 1, 2, 4, 7 ,14 and 21. The resonances at 7.9 and 8.9 ppm can most likely be assigned to different phosphonate decompositions products while the signal at 13.6 ppm is most likely caused by a species like **3** but with as cleaved phosphonate side arm.

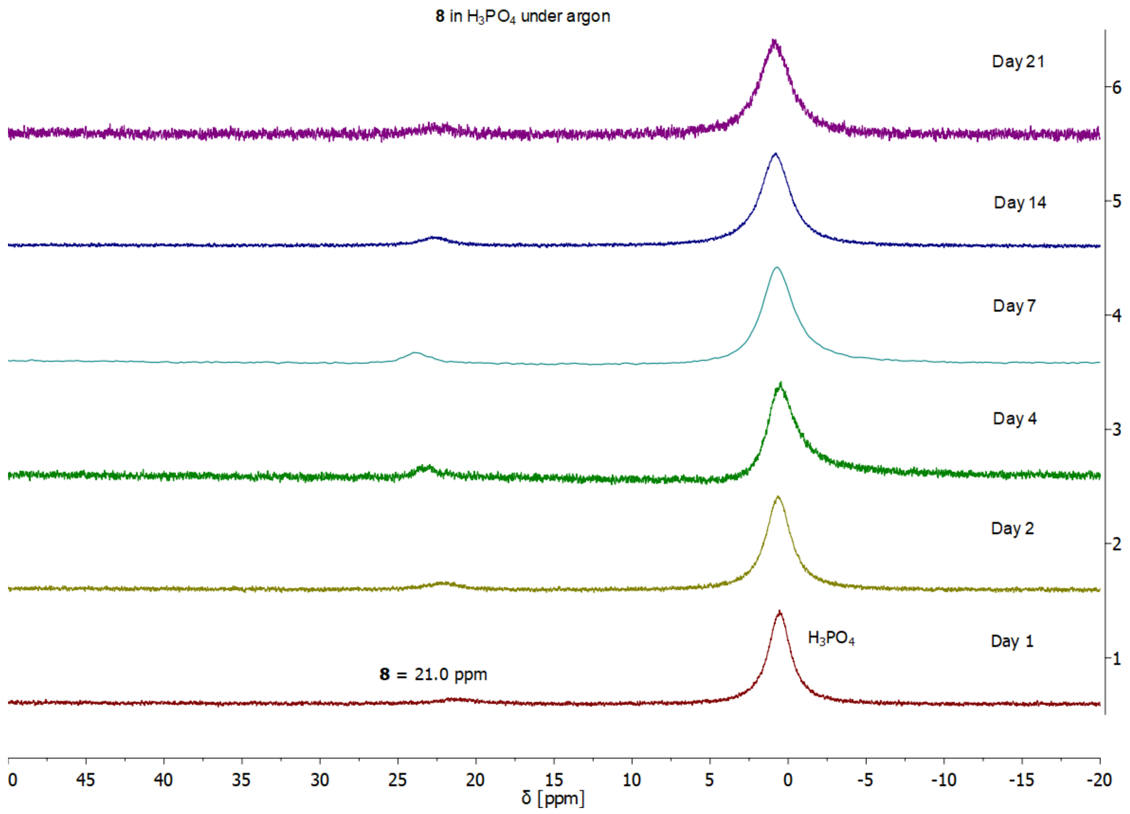

Figure S26. $^{31}P\{^{1}H\}$ NMR spectra of **8** in 0.5M H_3PO_4 under argon at day 1, 2, 4, 7 ,14 and 21. Pronounced line broadening due to the formation of strongly paramagnetic Fe(II) HS species.

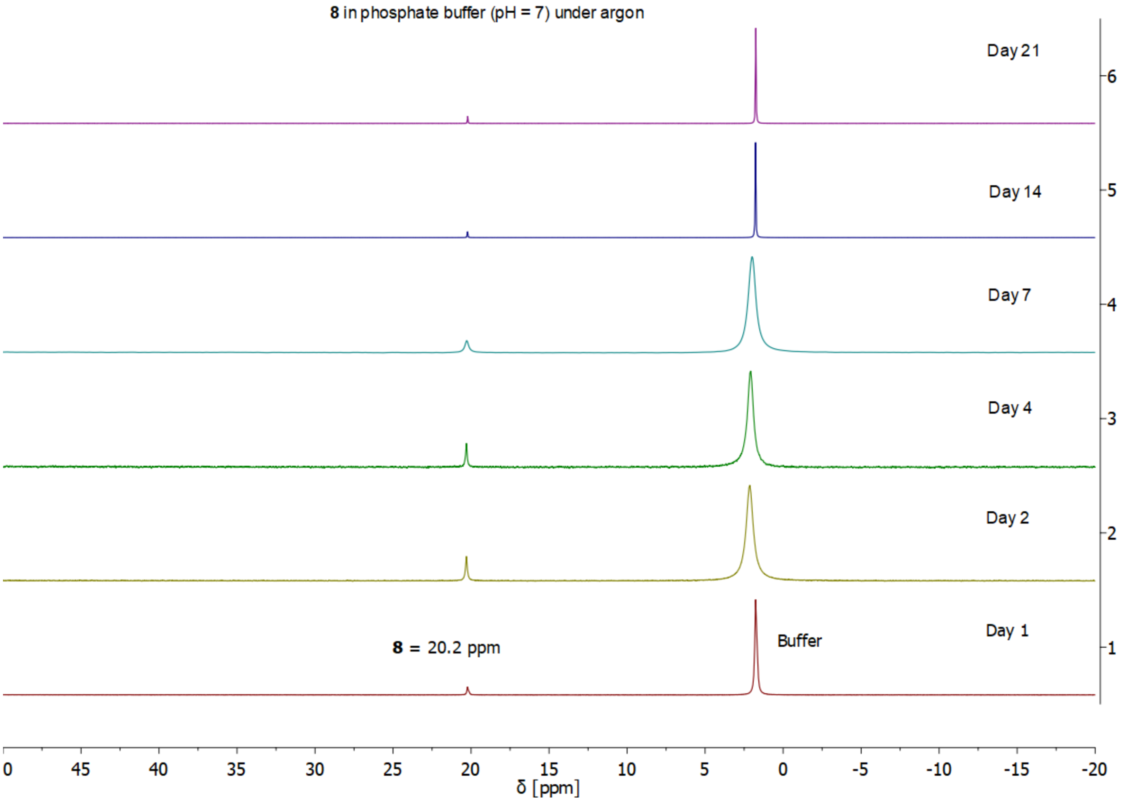

Figure S27. $^{31}P\{^{1}H\}$ NMR spectra of **8** in a phosphate buffer under argon at day 1, 2, 4, 7 ,14 and 21.

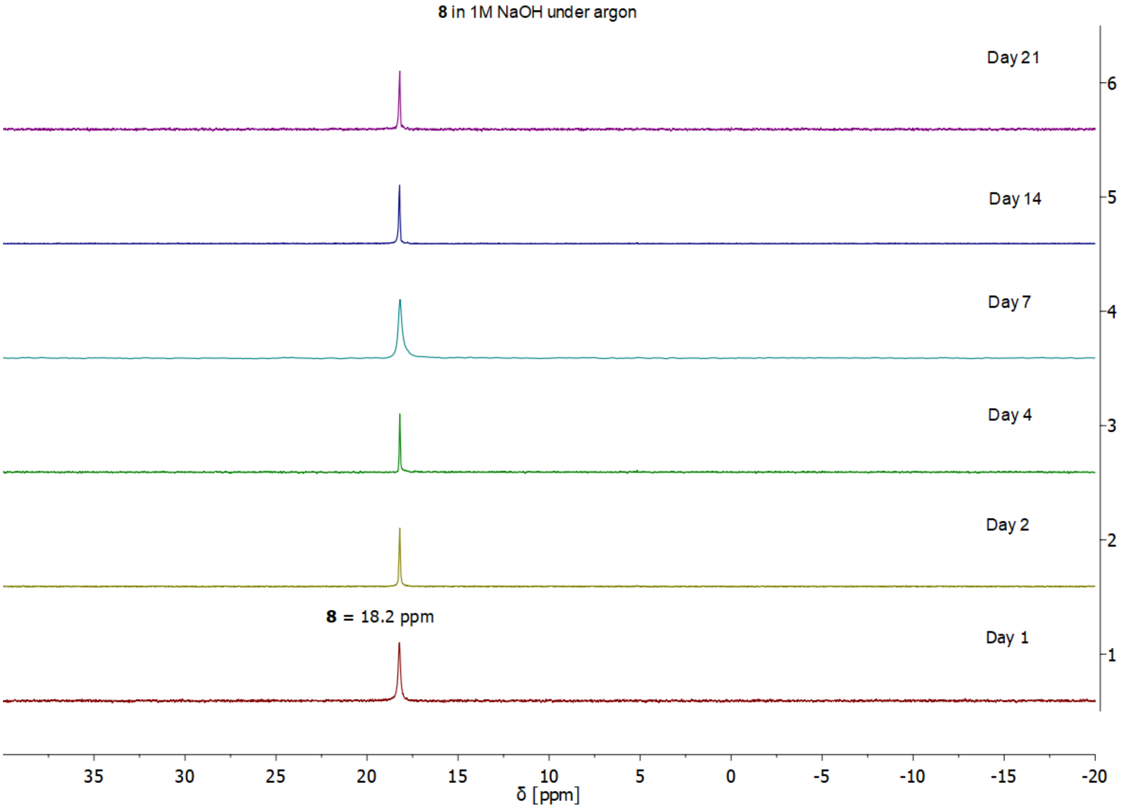

Figure S28. $^{31}P\{^{1}H\}$ NMR spectra of **8** in 1M NaOH under argon at day 1, 2, 4, 7 ,14 and 21.

5. IR-Spectra of the Precipitates from Solutions of 3 and 8 at Different pH Values

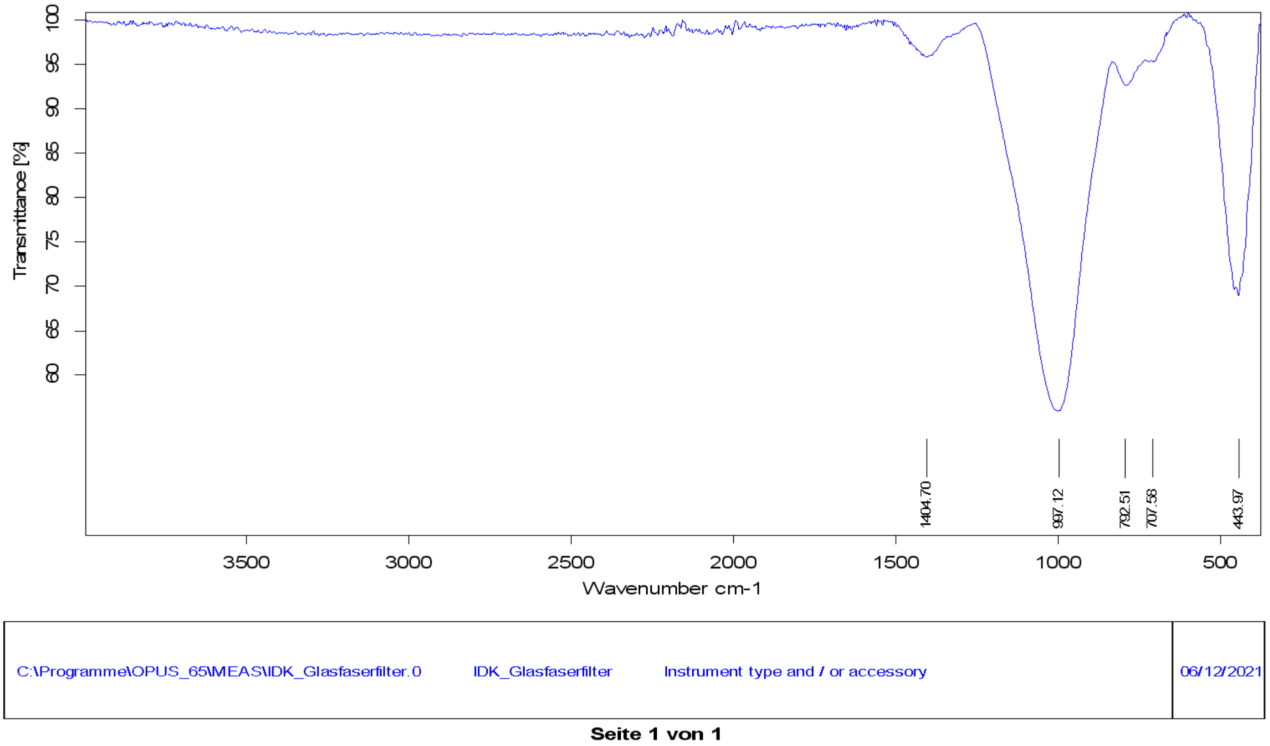

Figure S29. IR reference spectrum of the glass fibre filter material.

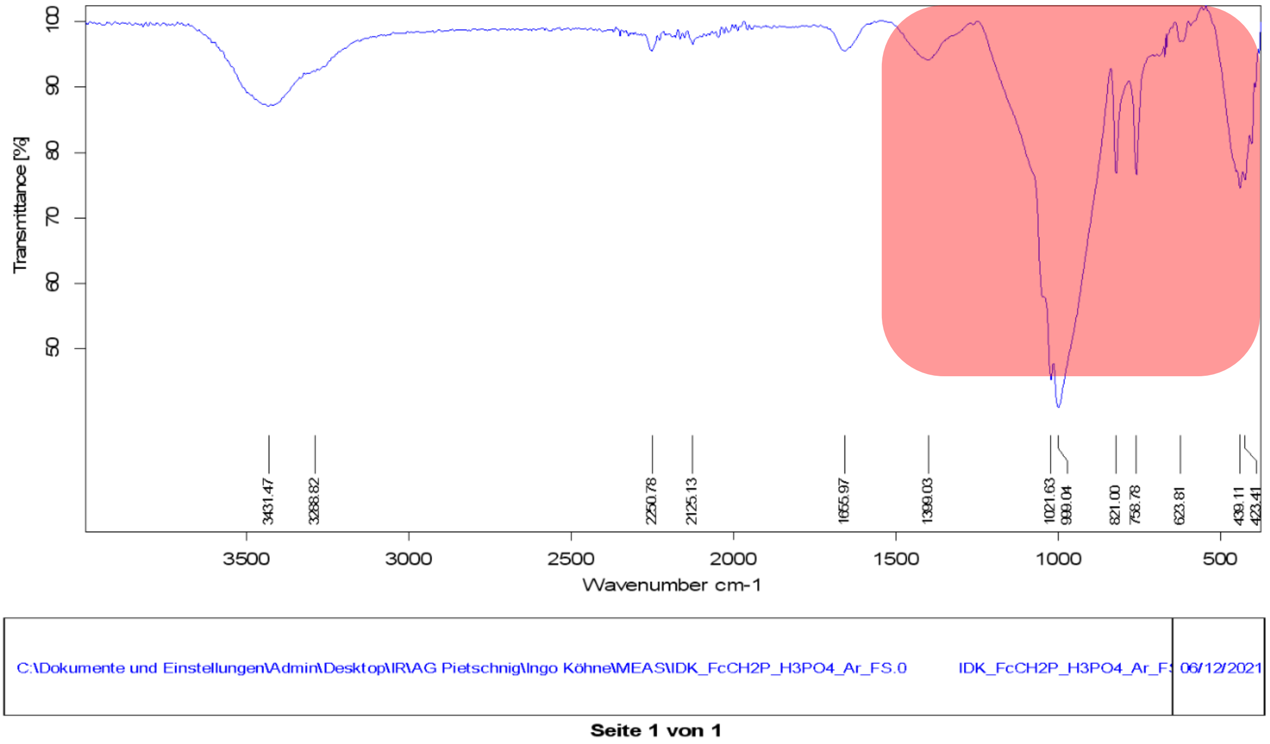

Figure S30. IR-spectrum of the Fe_2O_3 precipitate from a solution of **8** in 0.5M H_3PO_4 under argon. Bands belonging to a contamination with glass fiber from the filter material are highlighted in red.

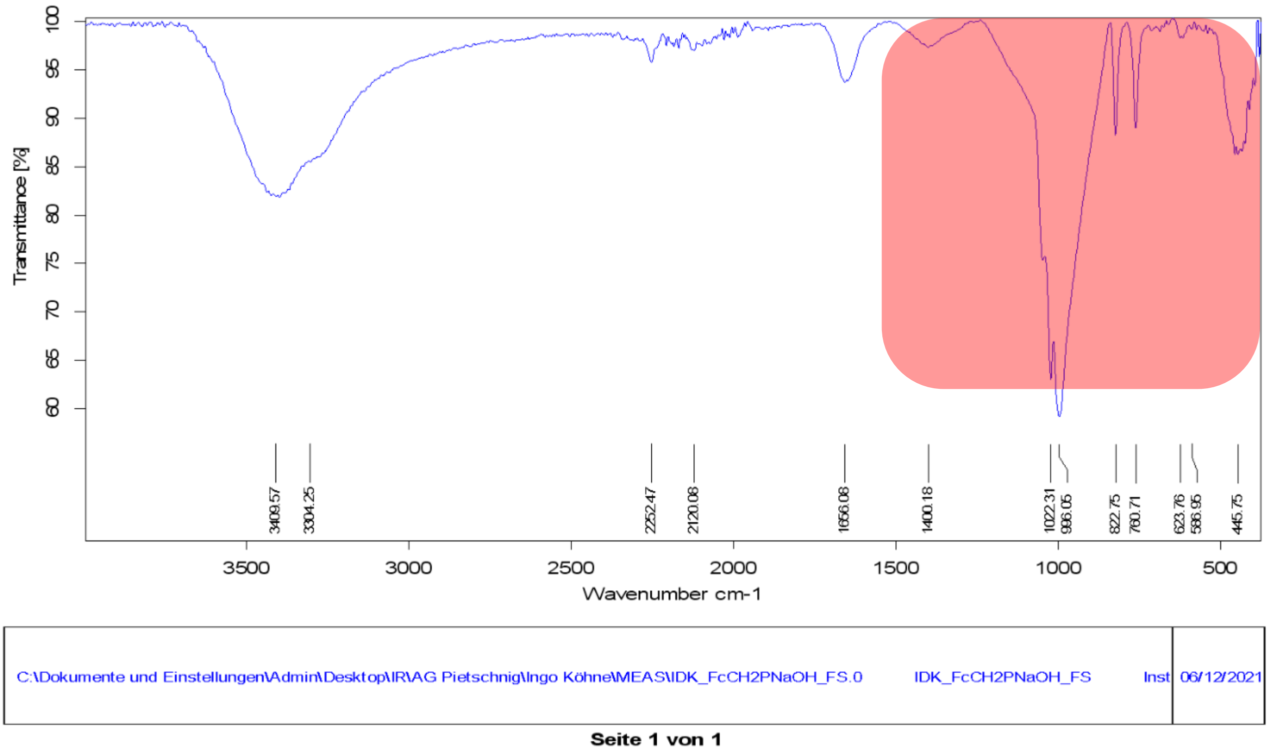

Figure S31. IR-spectrum of the Fe_2O_3 precipitate from a solution of **8** in 1M NaOH under air. Bands belonging to a contamination with glass fiber from the filter material are highlighted in red.

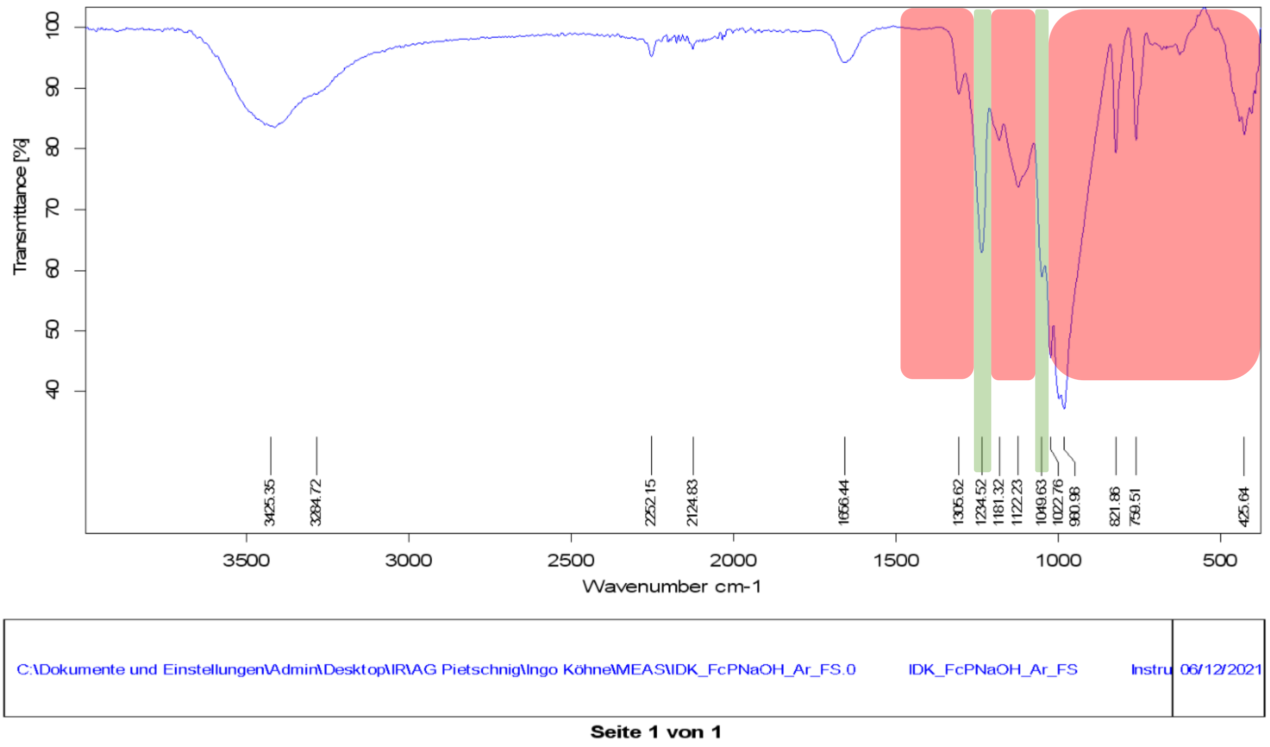

Figure S32. IR-spectrum of the Fe_2O_3 precipitate from a solution of **3** in 1M NaOH under argon. Bands belonging to a contamination with glass fiber from the filter material are highlighted in red. Bands highlighted in green might belong to a phosphonate decomposition product: P=O (1235 cm^{-1}) and P-O (1050 cm^{-1})

6. Pourbaix Plots

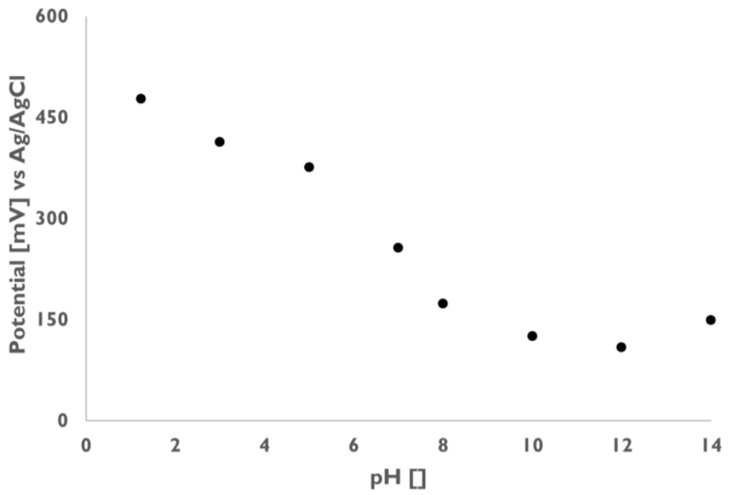

Figure S33. Pourbaix plot for **3**: 8 pH values between 1.23 and 14, c ≈ 0.5 mg/mL. CVs recorded at 100 mV/s. pH 1.23: 0.5 M H_3PO_4. pH 3: NaH_2PO_4/H_3PO_4, pH 5: $NaCH_3COO/CH_3COOH$, pH 7 and 8: NaH_2PO_4/Na_2HPO_4, pH 10 and 12: 0.5 M KCl + NaOH, pH 14: 1 M NaOH.

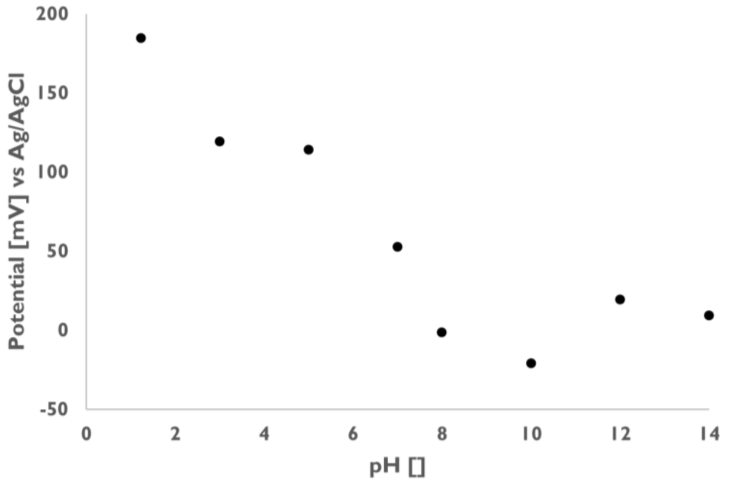

Figure S34. Pourbaix plot for **8:** 8 pH values between 1.23 and 14, c ≈ 0.5 mg/mL. CVs recorded at 100 mV/s. pH 1.23: 0.5 M H_3PO_4. pH 3: NaH_2PO_4/H_3PO_4, pH 5: $NaCH_3COO/CH_3COOH$, pH 7 and 8: NaH_2PO_4/Na_2HPO_4, pH 10 and 12: 0.5 M KCl + NaOH, pH 14: 1 M NaOH.

7. Randles-Sevcik Plots

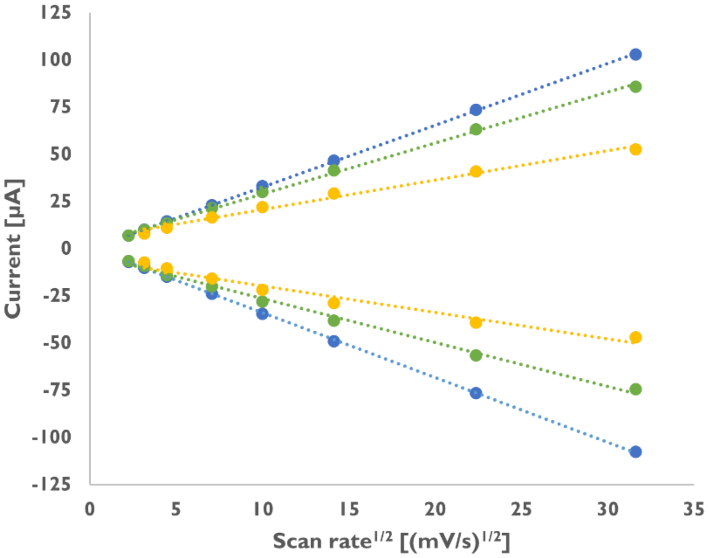

Figure S35. Randles-Sevcik plot for **3** at 3 different pH values with c ≈ 1.6 mg/mL. 8 scan rates between 1000 and 5 mV/s. Blue: pH 1.23 (0.5 M H_3PO_4), Green: pH 7 (0.5 M phosphate buffer solution), Yellow: pH 14 (1 M NaOH). pH 1.23: $I_{pa} = 3.28\ v^{-1/2} - 0.14$, $R^2 = 0.999$. $I_{pc} = -3.43\ v^{-1/2} + 0.23$, $R^2 = 0.999$. pH 7: $I_{pa} = 2.70\ v^{-1/2} - 1.88$, $R^2 = 0.998$. $I_{pc} = -2.32\ v^{-1/2} -3.33$, $R^2 = 0.995$. pH 14: $I_{pa} = 1.56\ v^{-1/2} - 5.09$, $R^2 = 0.990$. $I_{pc} = -1.40\ v^{-1/2} - 5.80$, $R^2 = 0.970$.

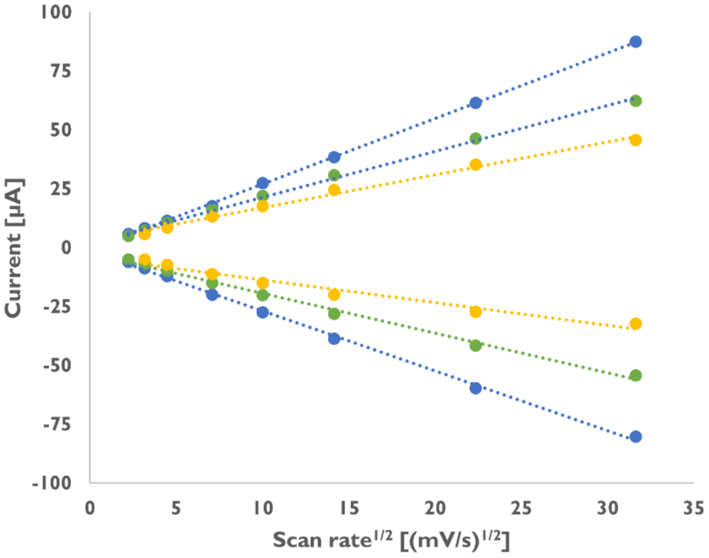

Figure S36. Randles-Sevcik plot for **8** at 3 different pH values with c ≈ 1.6 mg/mL. 8 scan rates between 1000 and 5 mV/s. Blue: pH 1.23 (0.5 M H_3PO_4), Green: pH 7 (0.5 M phosphate buffer solution), Yellow: pH 14 (1 M NaOH). pH 1.23: $I_{pa} = 2.79\ v^{-1/2} - 0.86$, $R^2 = 0.999$. $I_{pc} = -2.55\ v^{-1/2} - 1.24$, $R^2 = 0.998$. pH 7: $I_{pa} = 1.96\ v^{-1/2} - 1.89$, $R^2 = 0.998$. $I_{pc} = -1.69\ v^{-1/2} -2.46$, $R^2 = 0.995$. pH 14: $I_{pa} = 1.40\ v^{-1/2} - 3.08$, $R^2 = 0.992$. $I_{pc} = -0.96\ v^{-1/2} - 4.01$, $R^2 = 0.967$.

8. Sandwich and Full Flow Cell Experiments

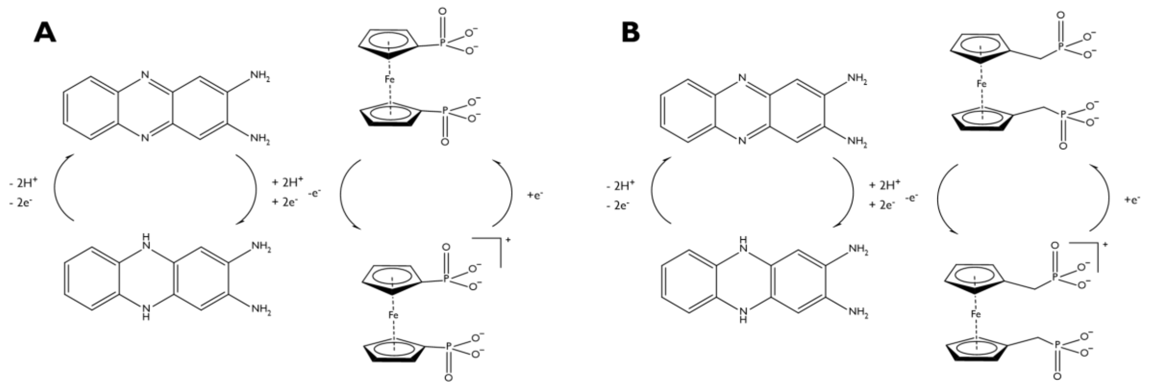

Figure S37. Scheme of the electrode reactions in the sandwich cell. Left: Negative electrode reaction, Right: Positive electrode reaction. **A**: Diaminophenazine *vs.* sodium 1,1'-ferrocene-bis(phosphonate) (**3**), **B**: Diaminophenazine *vs.* sodium 1,1'-ferrocene-bis(methylphosphonate) (**8**).

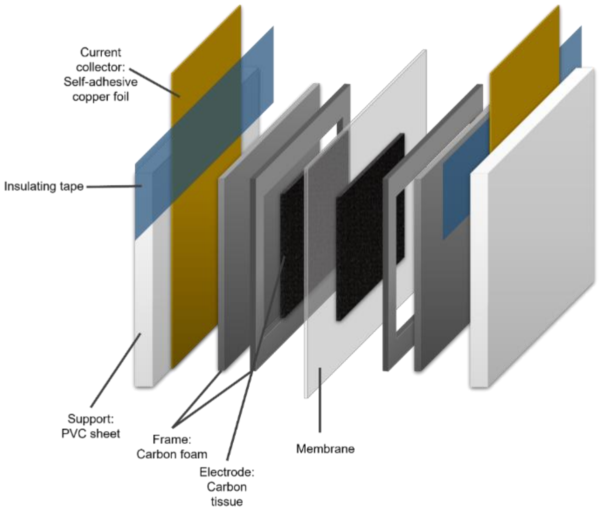

Figure S38. Schematic depiction of the sandwich cell.

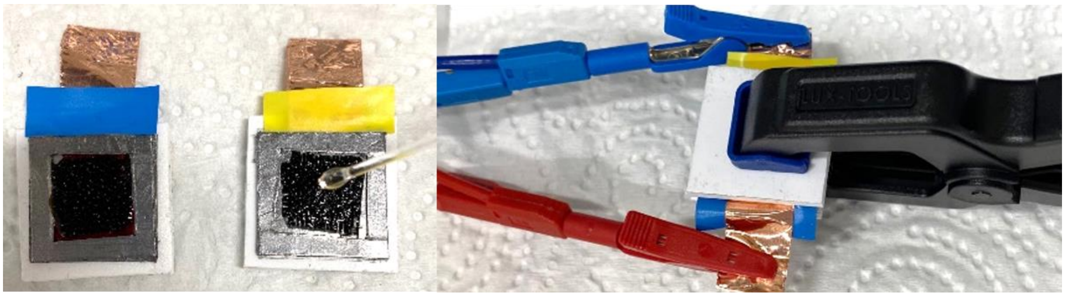

Figure S39. Sandwich cell.

Full flow battery tests were conducted in a flow battery cell with an active area of 6 cm^2, using a Nafion211 membrane and carbon felts from SignaCel. The battery was operated at a flow rate of 40 ml/min (Ismatec peristaltic pumps) and fully charged and discharged using current densities of 12 mA/cm^2. Concentration of anolyte and catholyte was 0.4 mM in 0.5 M H_3PO_4.

9. ESI-MS Spectra of the Filtrates of the ^{31}P-NMR Samples of 3 and 8

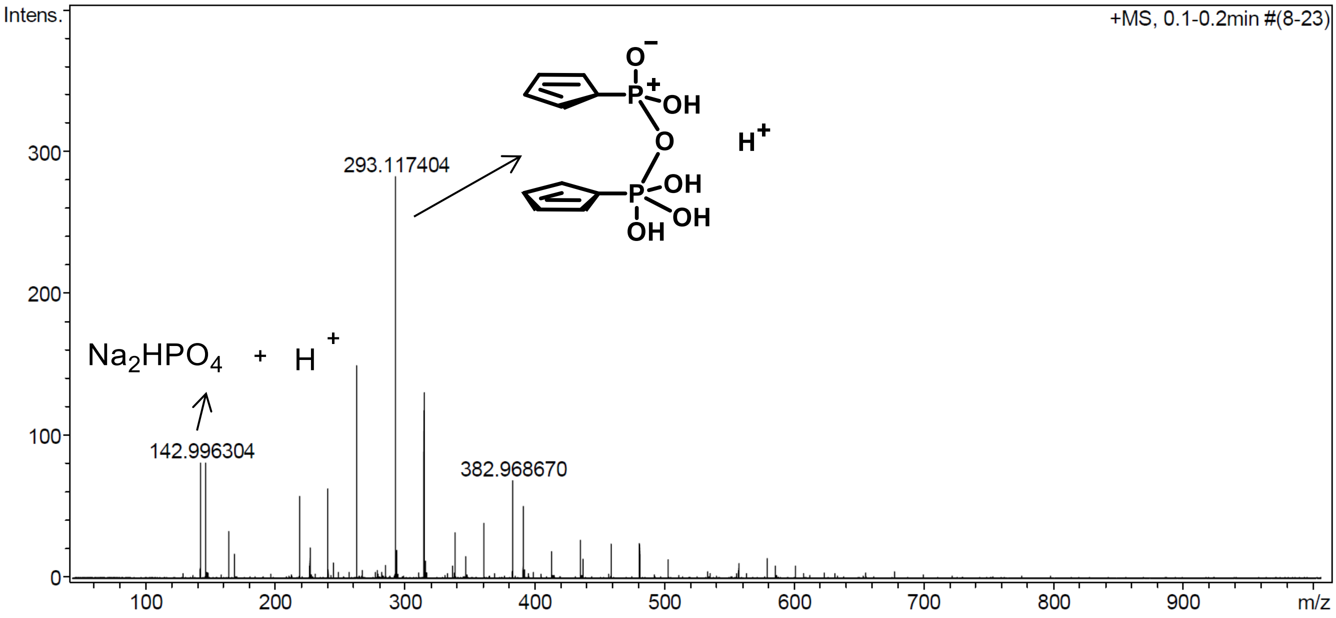

Figure S40. ESI-MS spectrum of the filtrate of **3** in a 0.5M H_3PO_4 solution under argon.

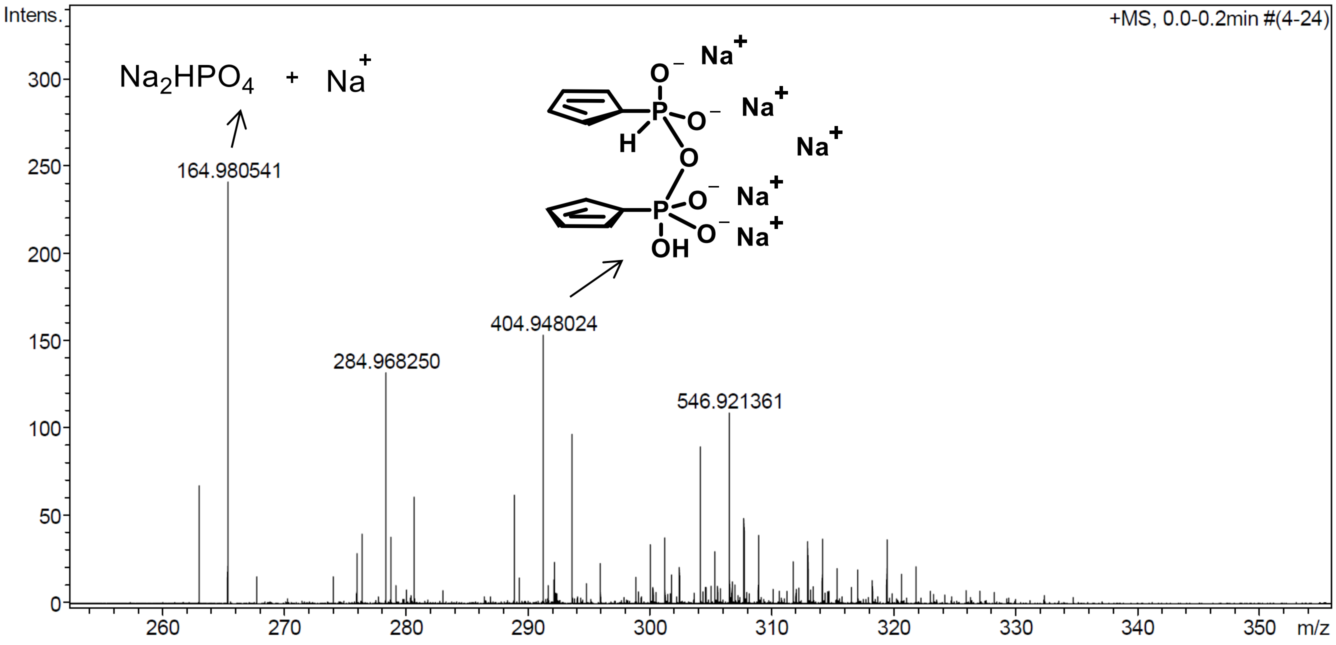

Figure S41. ESI-MS spectrum of the filtrate of **3** in a phosphate buffer (pH = 7) solution under air.

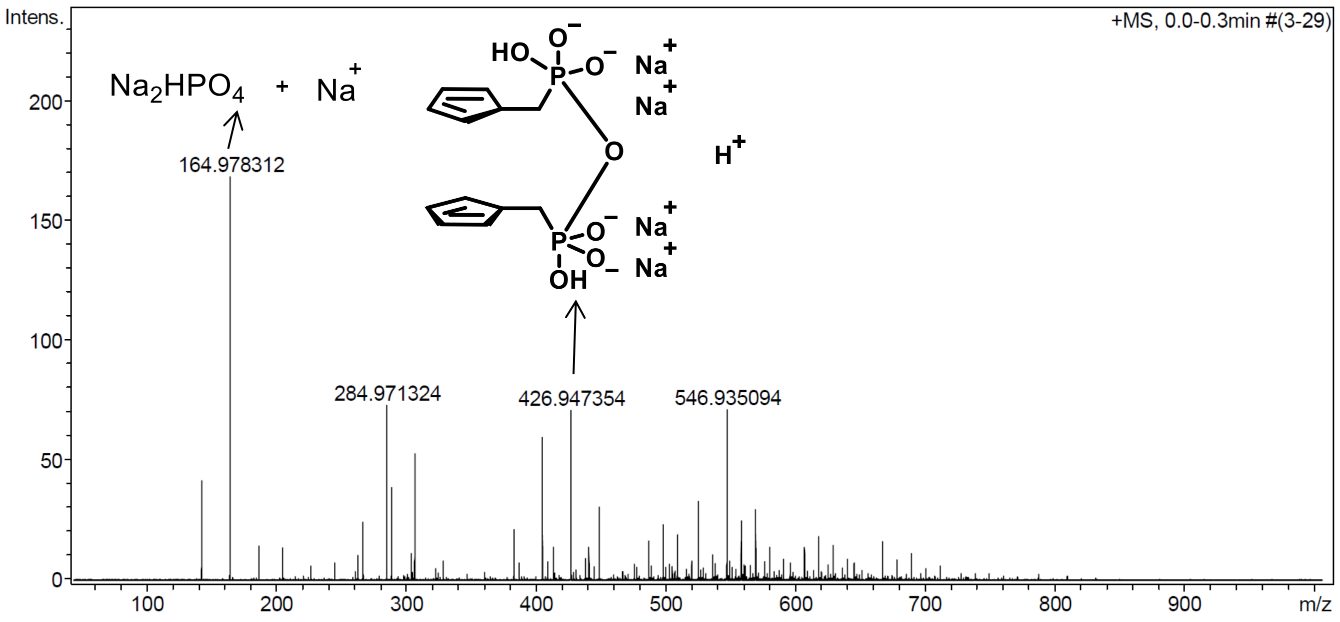

Figure S42. ESI-MS spectrum of the filtrate of **8** in a phosphate buffer (pH = 7) solution under air.

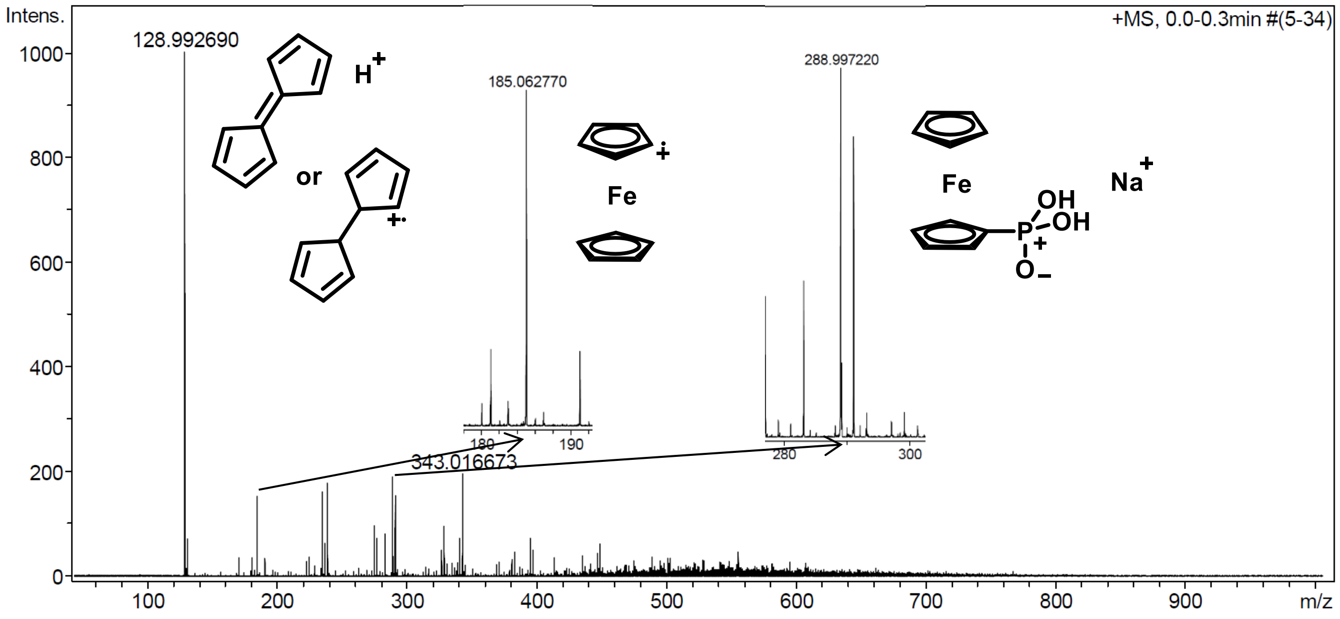

Figure S43. ESI-MS spectrum of the filtrate of **3** in a 1M NaOH solution under air.

10. Diffusion and Kinetic Parameters for the DAP and Fc Derivatives

Table S5. Summary of the diffusion and kinetic parameters for the DAP and ferrocene derivatives.

	Compound **3**	Compound **8**	DAP
$E^{0'}$ [V]	0.48	0.18	-0.18
ΔE [mV]	63	55	58
$D_{0,\,red}$ [$cm^2 \cdot s^{-1} \cdot 10^{-6}$][a]	2.30	1.51	1.91
$D_{0,\,ox}$ [$cm^2 \cdot s^{-1} \cdot 10^{-6}$][a]	2.10	1.69	1.98
D_0 [$cm^2 \cdot s^{-1} \cdot 10^{-6}$][b]	2.61	1.47	3.14
I_k [$A \cdot 10^{-4}$][b]	1.40	7.16	2.84
k_0 [$cm \cdot s^{-1} \cdot 10^{-3}$][b]	4.73	2.29	2.98
α[b]	0.65	0.57	0.51

[a]Randles-Sevcik analysis, [b]RDE

11. ^1H & $^{13}C\{^1H\}$ NMR Spectra of 3 & 8 after Aging Experiments under Argon in 0.5M H_3PO_4, Phosphate Buffer or 1M NaOH

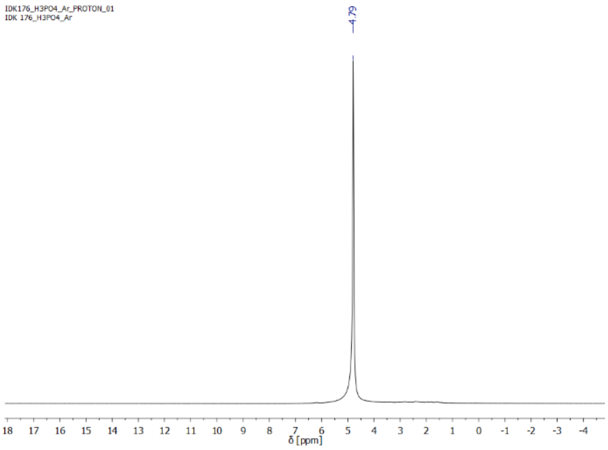

Figure S44. *^1H NMR spectrum of **3** in D_2O. Ageing in H_3PO_4 under argon. Signal belongs to D_2O.*

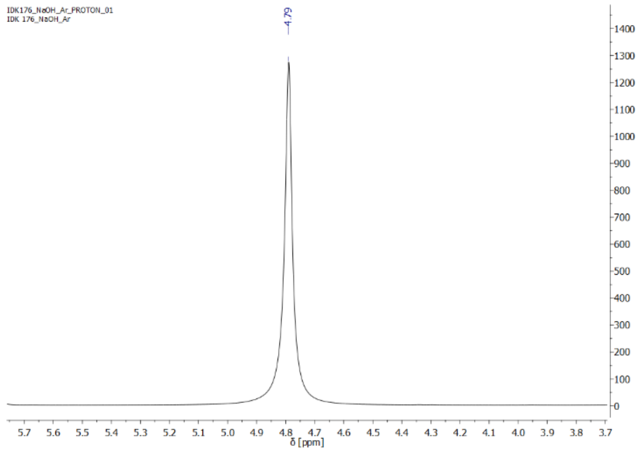

Figure S47. *^1H NMR spectrum of **3** in D_2O. Ageing in NaOH under argon. Signals belongs to D_2O.*

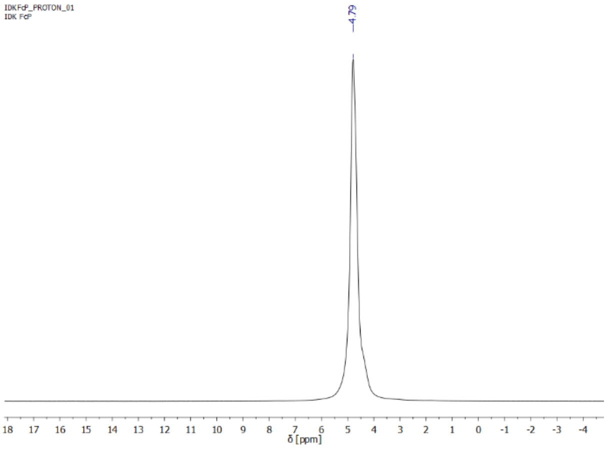

Figure S45. *^1H NMR spectrum of **3** in D_2O. Ageing in phosphate buffer under argon. Signal belongs to D_2O.*

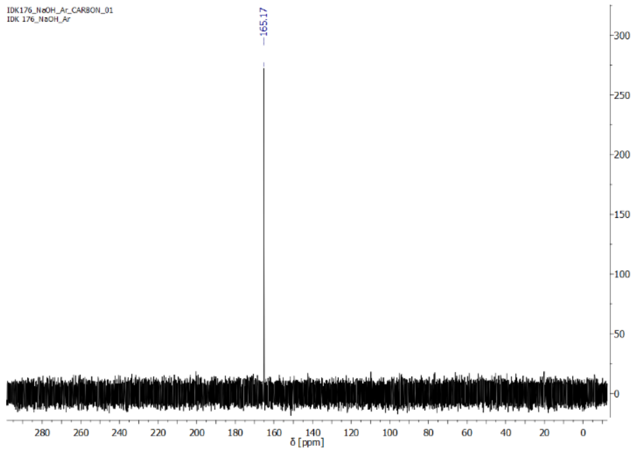

Figure S48. *$^{13}C\{^1H\}$ NMR spectrum of **3** in D_2O. Ageing in NaOH under argon. Signal belongs to an unknown species.*

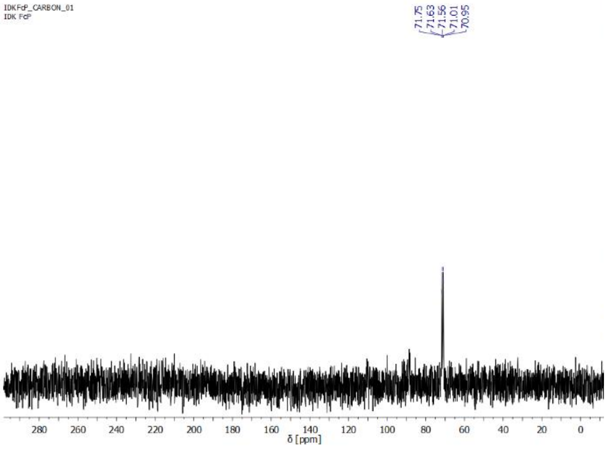

Figure S46. *$^{13}C\{^1H\}$ NMR spectrum of **3** in D_2O. Ageing in H_3PO_4 under argon. Signals belong to **3**.*

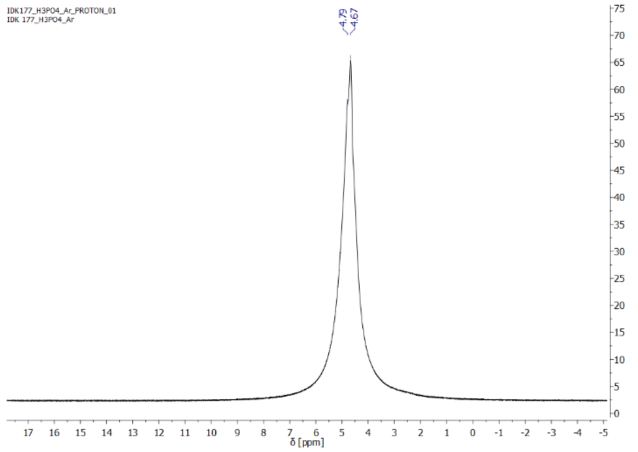

Figure S49. *^1H NMR spectrum of **8** in D_2O. Ageing in H_3PO_4 under argon. Signals belong to D_2O and **8**.*

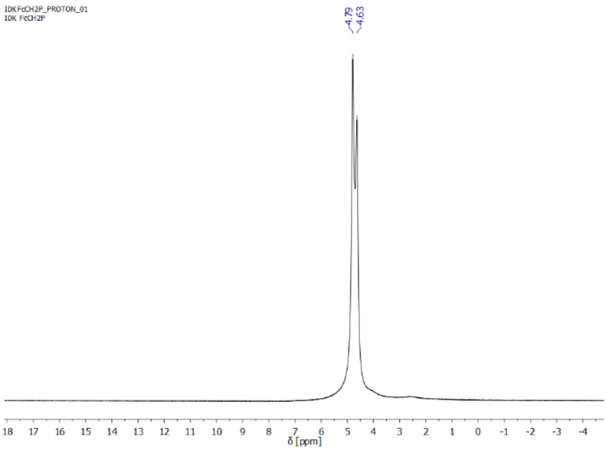

Figure S50. ^1H *NMR spectrum of* ***8*** *in* D_2O. *Ageing in phosphate buffer under argon. Signals belong to* D_2O *and* ***8***.

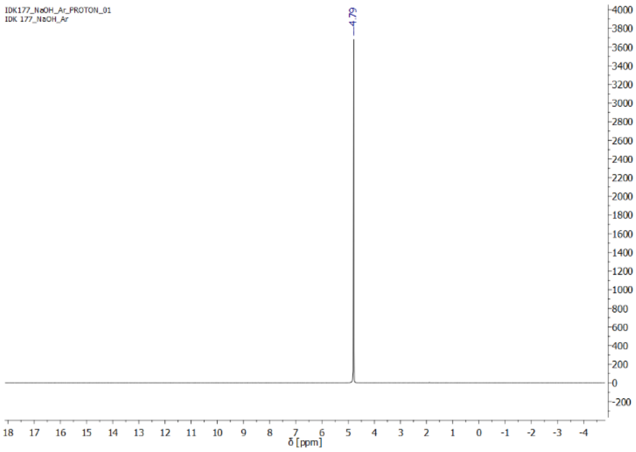

Figure S51. ^1H *NMR spectrum of* ***8*** *in* D_2O. *Ageing in NaOH under argon. Signal belongs to* D_2O.

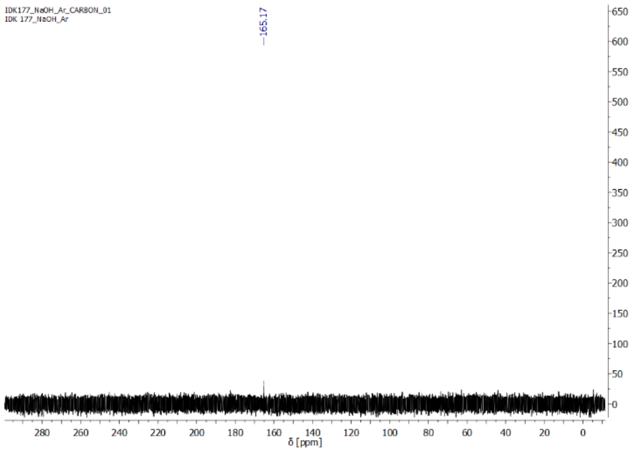

Figure S52. $^{13}C\{^1H\}$ *NMR spectrum of* ***8*** *in* D_2O. *Ageing in NaOH under argon. Signal belongs to an unknown species.*
